# Association between perinatal mortality and morbidity and customised and non-customised birthweight centiles in Denmark, Finland, Norway, Wales, and England: comparative, population based, record linkage study

**DOI:** 10.1136/bmjmed-2023-000521

**Published:** 2023-08-30

**Authors:** Fanny Kilpi, Hayley E Jones, Maria Christine Magnus, Gillian Santorelli, Lise Kristine Højsgaard Schmidt, Stine Kjaer Urhoj, Scott M Nelson, Derek Tuffnell, Robert French, Per Minor Magnus, Anne-Marie Nybo Andersen, Pekka Martikainen, Kate Tilling, Deborah A Lawlor

**Affiliations:** 1 MRC Integrative Epidemiology Unit, University of Bristol, Bristol, UK; 2 Population Health Sciences, Bristol Medical School, University of Bristol, Bristol, UK; 3 Centre for Fertility and Health, Norwegian Institute of Public Health, Oslo, Norway; 4 Bradford Institute for Health Research, Bradford, UK; 5 Department of Public Health, University of Copenhagen, Copenhagen, Denmark; 6 School of Medicine, Dentistry and Nursing, University of Glasgow, Glasgow, UK; 7 NIHR Bristol Biomedical Research Centre, University Hospitals Bristol NHS Foundation Trust and University of Bristol, Bristol, UK; 8 Cardiff University School of Medicine, Cardiff, UK; 9 Population Research Unit, University of Helsinki Faculty of Social Sciences, Helsinki, Uusimaa, Finland; 10 Max Planck Institute for Demographic Research, Rostock, Germany

**Keywords:** neonatology, obstetrics, perinatology

## Abstract

**Objectives:**

To compare the risk of adverse perinatal outcomes according to infants who are born small for gestational age (SGA; <10th centile) or large for gestational age (LGA; >90th centile), as defined by birthweight centiles that are non-customised (ie, standardised by sex and gestational age only) and customised (by sex, gestational age, maternal weight, height, parity, and ethnic group).

**Design:**

Comparative, population based, record linkage study with meta-analysis of results.

**Setting:**

Denmark, Finland, Norway, Wales, and England (city of Bradford), 1986-2019.

**Participants:**

2 129 782 infants born at term in birth registries.

**Main outcome measures:**

Stillbirth, neonatal death, infant death, admission to neonatal intensive care unit, and low Apgar score (<7) at 5 minutes.

**Results:**

Relative to those infants born average for gestational age (AGA), both SGA and LGA births were at increased risk of all five outcomes, but observed relative risks were similar irrespective of whether non-customised or customised charts were used. For example, for SGA versus AGA births, when non-customised and customised charts were used, relative risks pooled over countries were 3.60 (95% confidence interval 3.29 to 3.93) versus 3.58 (3.02 to 4.24) for stillbirth, 2.83 (2.18 to 3.67) versus 3.32 (2.05 to 5.36) for neonatal death, 2.82 (2.07 to 3.83) versus 3.17 (2.20 to 4.56) for infant death, 1.66 (1.49 to 1.86) versus 1.54 (1.30 to 1.81) for low Apgar score at 5 minutes, and (based on Bradford data only) 1.97 (1.74 to 2.22) versus 1.94 (1.70 to 2.21) for admission to the neonatal intensive care unit. The estimated sensitivity of combined SGA or LGA births to identify the three mortality outcomes ranged from 31% to 34% for non-customised charts and from 34% to 38% for customised charts, with a specificity of 82% and 80% with non-customised and customised charts, respectively.

**Conclusions:**

These results suggest an increased risk of adverse perinatal outcomes of a similar magnitude among SGA or LGA term infants when customised and non-customised centiles are used. Use of customised charts for SGA/LGA births—over and above use of non-customised charts for SGA/LGA births—is unlikely to provide benefits in terms of identifying term births at risk of these outcomes.

**WHAT IS ALREADY KNOWN ON THIS TOPIC**

- Use of thresholds for infants born small for gestational age (<10th centile) and large for gestational age (>90th centile) can help detect fetal growth problems
- Fetal growth problems can identify fetuses at a higher risk of stillbirth, neonatal mortality, and severe morbidity

**WHAT THIS STUDY ADDS**

- Incorporating information on maternal weight, height, parity, and ethnic group to define term births that are small or large for gestational age might not have a discernible benefit for detecting associations with perinatal mortality or morbidity, compared with standardising for gestational age and infant sex only
- These results provide further evidence that the added benefits of using customised charts for these outcomes in clinical practice are limited

**HOW THIS STUDY MIGHT AFFECT RESEARCH, PRACTICE, OR POLICY**

- Widespread adoption of customised centile charts should not be promoted

## Introduction

Fetal growth problems underlie a high proportion of stillbirths, neonatal mortality, and severe morbidity.^1–5^ An important aim of antenatal care is the detection and management of such problems to minimise adverse outcomes.^6 7^ Birthweight centile charts aim to reflect healthy (or physiological) growth and these are converted to estimated fetal growth charts used antenatally. Traditionally, such charts control for sex and gestational age because these factors are seen as physiological aspects of fetal growth. However, customised charts adjust for additional characteristics—maternal height, weight, parity, and ethnic group—considering that these factors reflect physiological rather than pathological differences in fetal growth.^8 9^ Customised charts aim to indicate the optimal fetal growth potential, against which maternal fundal height measurements and estimated fetal weight (from ultrasound scan) might be plotted throughout pregnancy.

In a systematic review and meta-analysis that included up to 20 studies, with up to 1 095 589 pregnancies, researchers concluded that both customised and non-customised charts identified increased risk of adverse outcomes, particularly for small-for-gestational-age (SGA) infants, but had insufficient power to robustly determine the difference between the two.^10^ Individual studies published since have differed in size from 4095 to 1.25 million and have come to varying conclusions. Three of these studies, a record linkage study of about 1 million births from Scotland, a UK pregnancy cohort of about 4000 births to nulliparous women, and a Swedish record linkage study of over 200 000 births, did not find evidence of differences between non-customised and customised charts in predicting adverse perinatal outcomes in singleton births.^2 11 12^ By comparison, a study of 1.25 million singleton term births from 10 countries and a study of about 53 000 singleton pregnancies from New Zealand found that customised centile charts identified more SGA infants and more of those who were at risk of stillbirth than the Intergrowth-21st standards.^13 14^

These previous studies have several limitations. The large Scottish record linkage study did not have information on maternal ethnic group or weight and was only able to partially customise.^2^ This limitation is important because the other large study^13^ and the smaller New Zealand study^14^ suggested that better prediction with customised charts compared with Intergrowth-21st charts could reflect that Intergrowth-21st charts do not not account for the physiological effect of maternal ethnic group on fetal growth. However, the larger of those two studies only explored stillbirth as an outcome,^13^ and the smaller study had a composite neonatal outcome combining mortality and morbidity.^14^

The aim of our study was to compare how SGA and large-for-gestational-age (LGA) infants defined according to customised birthweight centiles (by fetal sex, gestational age, maternal weight, height, parity, and maternal ethnic group) and non-customised birthweight centiles (adjusted for fetal sex and gestational age only) are associated with perinatal mortality and morbidity. We used record linkage data from Denmark, Finland, Norway, Wales, and England, and explored consistency of findings across different populations.

## Methods

### Data

We used record linkage data from five countries: national data from Denmark (2004-10), Finland (2004-14), Norway (2012-16), Wales (1986-2016), and regional data from the city of Bradford in England (2010-19). Full details of each dataset are available in the online supplemental material. Information from the separate countries was harmonised, and the analysis sample was restricted to singleton births that occurred between 37 and 43 gestational weeks, as in most previous studies, to distinguish fetal growth problems from preterm birth effects. We excluded infants with known major congenital anomalies as causes of death or identified within the first year of life to exclude cases where congenital anomalies might be the cause of fetal growth restriction, and influenced by some of the customised variables (eg, body mass index). We excluded observations with missing data on sex, birthweight or gestational age at delivery, and with birthweight outside the 0.15-8.00 kg range. We also excluded observations with customisation variables outside the ranges previously suggested for customised charts (maternal height 100-213 cm, maternal pre-pregnancy weight or early pregnancy weight 30-300 kg, parity 0-15).^15^ Online supplemental table S1 describes the sample selection (overall number of participants excluded: Bradford 3919 (8%), Denmark 49 437 (11%), Finland 73 715 (11%), Norway 17 628 (6%), Wales 94 327 (10%)).

10.1136/bmjmed-2023-000521.supp1 Supplementary data

### Predictors

#### Non-customised birthweight centiles

As in most previous research, birth weight was used as a proxy to reflect fetal growth across pregnancy. We generated non-customised birthweight centiles that were standardised for infant sex and gestational age using the UK 1990 reference population within the zanthro package in Stata to derive z scores, and then created centiles within countries from the ordering of those scores. For both customised and non-customised centiles, we categorised infants into the following groups: SGA (<10th centile), average-for-gestational age (AGA; 10th-90th centile) and LGA (>90th centile).

#### Customised birthweight centiles

In addition to infant sex and gestational age, the variables used for customisation are maternal ethnic group, height, weight, and parity.^15^ Nordic countries in general collect information on country of birth or origin but not self-reported ethnic group. Thus, we based ethnic group on the mother's self-reported ethnic group for Bradford and Wales and on the mother's country of birth or origin for Denmark, Finland, and Norway. Ethnic group categories with small numbers of observations (<100) were collapsed into higher category levels. For example, if a dataset had fewer than 100 pregnancies in women of Bangladeshi origin they would be classified as South Asian, or as "other" if the number of South Asian participants was still <100. Maternal height was reported in centimetres (range 100-213 cm); maternal pre-pregnancy or early pregnancy weight in kilograms (30-300 kg); and parity categorised as zero, one, two, three, and four or more.

We used the Perinatal Institute's global centile bulk calculator (version 8.0.4) to calculate customised centiles in Bradford data.^15^ Use of the calculator required inputting data into an external server, which was not feasible for other countries owing to data governance reasons, so the customised centiles for the other countries were calculated by use of equations based on the methods published by the Perinatal Institute.^16^ Customised birthweight centiles were generated within each dataset by first regressing birth weight on gestational age at delivery, infant sex, maternal height, weight, parity, and ethnic group in complete case data (ie, no missing data on variables used in the customisation). We then calculated a predicted birth weight for all observations based on gestational age, sex, and available maternal characteristics. If a customisation variable was missing, we assumed median weight or height, majority ethnic group, or parity=0 for the purpose of these predictions. Following this, we calculated whether the observed birth weight fell within the calculated customised SGA and LGA thresholds for each observation's predicted birth weight. Full details of the methods are provided in supplementary methods and the customised coefficients for each dataset in online supplemental table S2.

### Outcomes

We examined the following outcomes in all countries when available: stillbirth (birth of an infant without signs of life), neonatal death (death within first 28 days of life for liveborn infants), infant death (death within first year of life for liveborn infants), low Apgar score at 5 minutes for livebirths (score <7, which includes 4-6=moderately abnormal and 0-3=low, to increase power and precision), and admission to the neonatal intensive care unit (NICU) within the first week of life.

### Analysis strategy

#### Main analyses

We used log binomial regression to separately estimate the relative risk of each outcome comparing SGA and LGA births with AGA births using the non-customised and customised centiles in each country. We meta-analysed the results across the five countries with a restricted, maximum likelihood, random effects model using the meta command in Stata (version 16). An SGA or LGA classification alters obstetric care pathways, so we also calculated the sensitivity and specificity of combined SGA/LGA births versus AGA births to predict each outcome again, based on non-customised and on customised centiles. Sensitivity is calculated by the number of outcome cases identified as SGA/LGA births divided by the total number of outcome cases (ie, true positives/(true positives+false negatives)), and specificity by the number of cases without the outcome identified as AGA divided by the total number of cases without the outcome (ie, true negatives/(true negatives+false positives)). We used the command metandi^17^ to perform bivariate random effects meta-analysis of sensitivity and specificity for each outcome.

#### Supplementary analyses

We performed several sensitivity analyses. Firstly, we explored the sensitivity of the results from each dataset to their main sources of missing data either by using more years of data available with only partial customisation (eg, only customising for ethnic group and parity in data from Denmark and Norway) or by checking results when using more restricted datasets with less missingness in customisation variables (Bradford and Wales) (detailed in the supplementary methods). Secondly, we meta-analysed data from the three Nordic countries separately from the two UK datasets, on the basis that information on ethnic group was collected differently between these countries. Thirdly, at the suggestion of a reviewer, we performed a meta-analysis excluding data from Norway because we were not able to exclude those with congenital anomalies in these data. Finally, to explore whether generating our own customised charts within each study might have influenced results, we repeated analyses for Bradford using within-study customisation equations and compared the results with those in the main analyses where we used the bulk calculator for customisation.

### Patient and public involvement

The current research uses secondary data and was not informed by patient and public involvement. No patients were involved in setting the research question, developing the study design or analysis plan, or setting the outcomes measures. The results will be disseminated to stakeholders and the broader public as relevant. There are no plans to disseminate the results of the research to individual study participants because all participants are deidentified.

## Results

The analysis included 2 129 782 term births, with 191 923 identified as SGA and 212 732 as LGA with non-customised centiles, and 215 719 as SGA and 217 836 as LGA with customised centiles. Table 1 details the characteristics of the five datasets. Median birth weight was lowest in Bradford (3300 g) and highest in Denmark and Finland (3570 g). The proportions of stillbirth, neonatal, and infant death were highest in Bradford (0.24%, 0.10%, and 0.20%, respectively), and low Apgar score at 5 minutes had highest prevalence in Wales (3.25%). The Welsh data had the highest proportion of nulliparous mothers (45%), and the Bradford data had the lowest (33%).

**Table 1:** Descriptive characteristics of study populations

	Bradford, England (n=47 583)	Denmark (n=384 885)	Finland (n=576 758)	Norway (n=276 078)	Wales (n=844 478)
Years	2010-19	2004-10	2004-14	2012-16	1986-2016
Median (IQR) birth weight (g)	3300 (2990-3630)	3570 (3250-3900)	3570 (3270-3890)	3570 (3260-3890)	3430 (3120-3760)
Median (IQR) gestational age (days)	279 (273-285)	281 (274-287)	281 (275-287)	281 (275-287)	280 (273-287)
Outcomes (No (%))					
Stillbirth	112 (0.24)	516 (0.13)	538 (0.09)	370 (0.13)	1102 (0.13)
Neonatal death	47 (0.10)	236 (0.06)	139 (0.02)	129 (0.05)	244 (0.03)
Infant death	93 (0.20)	365 (0.09)	351 (0.06)	242 (0.09)	625 (0.07)
Apgar score <7 at 5 minutes	427 (0.90)	1874 (0.49)	7498 (1.53)	2699 (0.98)	19 030 (3.25)
NICU admission	1849 (3.89)	NA	NA	NA	NA
Maternal characteristics					
Minority ethnic group (No (%))	21 126 (44)	53 609 (14)	52 532 (9)	75 421 (28)	29 875 (8)
Missing information on ethnic group (%)	26	<1	<1	1	53
Median (IQR) height (cm)	162 (157-166)	168 (164-172)	165 (162-170)	167 (163-171)	163 (159-168)
Missing information on height (%)	8	6	2	26	53
Median (IQR) weight (kg)	66 (58-76)	65 (59-75)	64 (57-73)	65 (58-74)	64 (57-74)
Missing information on weight (%)	6	6	5	29	54
No previous births (No (%))	15 748 (33)	164 484 (43)	238 462 (41)	115 763 (42)	320 532 (45)
Missing information on parity (%)	1	0	<1	0	15

IQR=interquartile range; NICU=neonatal intensive care unit; NA=not available.

*Assigned “global average” in customisation main analyses.

**Figure 1:**
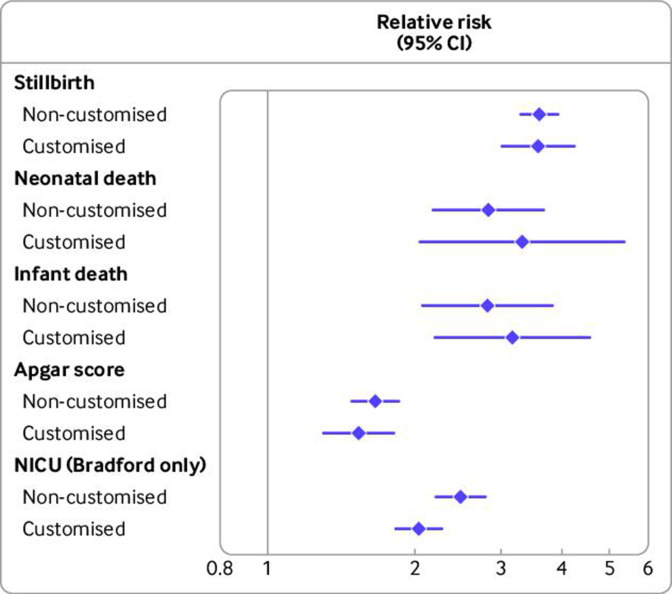
Pooled risk ratio estimates for perinatal adverse outcomes by small-for-gestational-age (<10th centile) births versus average-for-gestational-age (10-90th centile) births with non-customised and customised birthweight centiles (n=2 129 782 births, 1986-2019). Pooled results are from Bradford, England (n=47 583, 2010-19), Denmark (n=384 885, 2004-10), Finland (n=576 758, 2004-14), Norway (n=276 078, 2012-16), and Wales (n=844 478, 1986-2016). Numbers of cases/births for outcomes: stillbirth 2683/2 129 782, neonatal death 795/2 127 697, infant death 1676/2 127 697, low Apgar score at 5 minutes 31 633/1 780 158, NICU admission 1849/47 471. NICU=neonatal intensive care unit; error bars=95% confidence intervals

Figure 1 presents the pooled relative risk of each adverse perinatal outcome for infants born SGA versus AGA, as defined by non-customised and customised definitions (online supplemental figure S1 includes meta-analysis forest plots, and online supplemental table S3A-E includes detailed country specific estimates). For stillbirth (relative risk 3.60 (95% confidence interval 3.29 to 3.93) *v* 3.58 (3.02 to 4.24) for non-customised and customised SGA births, respectively), neonatal death (2.83 (2.18 to 3.67) *v* 3.32 (2.05 to 5.36)), infant death (2.82 (2.07 to 3.83) *v* 3.17 (2.20 to 4.56)), and low Apgar score (1.66 (1.49 to 1.86) *v* 1.54 (1.30 to 1.81)), the pooled relative risks were consistent for non-customised and customised definitions of SGA. In Bradford (the only study with data on NICU admission), the relative risk of admission to NICU was 2.48 (2.20 to 2.79) with SGA based on non-customised centiles and 2.04 (1.83 to 2.27) based on customised centiles (online supplemental table S3A). In Danish analyses, customised SGA had consistently higher relative risks than non-customised SGA for all outcomes, while the reverse was the case for the Bradford analysis. In other countries, the results were more mixed. We saw varying heterogeneity in the pooled analyses, with heterogeneity statistics up to I^2^=94.10% for the analyses of Apgar score by customised SGA.

**Figure 2:**
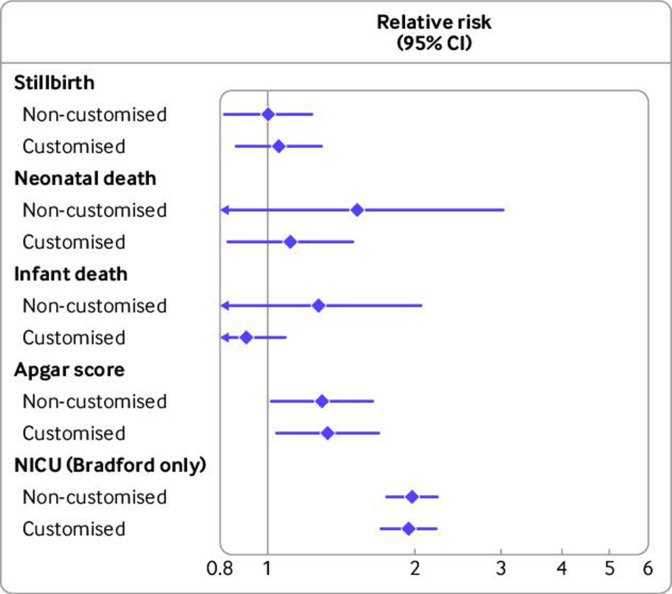
Pooled risk ratio estimates for perinatal adverse outcomes by large-for-gestational-age (>90th centile) births versus average-for-gestational-age (10-90th centile) births with non-customised and customised birthweight centiles (n=2 129 782 births, 1986-2019). Pooled results are from Bradford, England (n=47 583, 2010-19), Denmark (n=384 885, 2004-10), Finland (n=576 758, 2004-14), Norway (n=276 078, 2012-16), and Wales (n=844 478, 1986-2016). Number of cases/births for outcomes: stillbirth 2683/2 129 782, neonatal death 795/2 127 099, infant death 1676/2 127 099, low Apgar score at 5 minutes 31 633/1 780 158, NICU admission 1849/47 471. NICU=neonatal intensive care unit; error bars=95% confidence intervals

Figure 2 shows the pooled relative risks by non-customised and customised definitions of LGA versus AGA births (online supplemental figure S2 shows meta-analysis forest plots and online supplemental table S3A-E shows detailed study specific estimates). For stillbirth (non-customised, relative risk 1.00 (95% confidence interval 0.82 to 1.23) *v* customised, 1.05 (0.86 to 1.29)), results for both charts were close to the null and consistent with each other. By contrast, for neonatal death (1.54 (0.76 to 3.03) *v* 1.11 (0.83 to 1.49)) and infant death (1.27 (0.78 to 2.07) *v* 0.90 (0.75 to 1.09)), the relative risk was greater with the non-customised definition of LGA than for customised LGA, although for both the confidence intervals were wide and included the null value. For Apgar score (1.29 (1.02 to 1.64) *v* 1.33 (1.04 to 1.69)) and NICU admission (in Bradford only) (1.97 (1.74 to 2.22) *v* 1.94 (1.70 to 2.21)), LGA birth was associated with increased risk to a similar magnitude with non-customised and customised centiles. Neither customised nor non-customised LGA births was consistently associated with increased risk of one outcome compared with the other outcomes in any country. Greatest amount of heterogeneity was found for Apgar score by customised definitions of LGA births (I^2^=97.07%).

**Figure 3:**
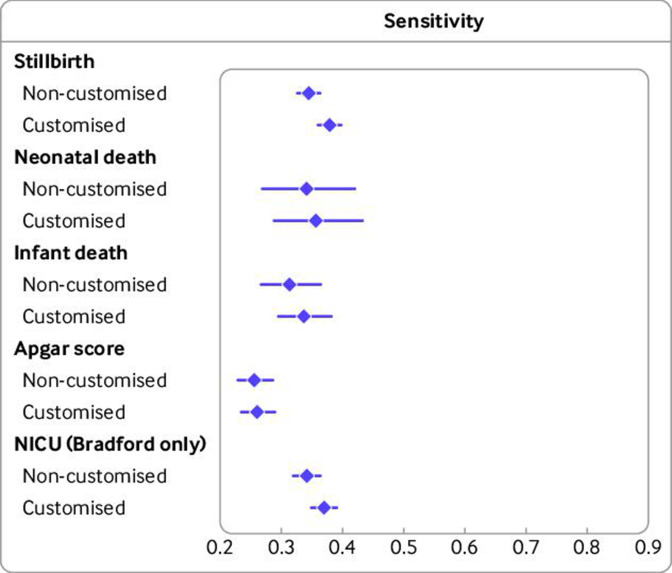
Pooled sensitivity of small-for-gestational-age (<10th age) and large-for-gestational-age (>90th centile) births versus average-for-gestational-age (10-90th centile) births for adverse perinatal outcomes with non-customised and customised birthweight centiles (n=2 129 782 births, 1986-2019). Pooled results are from Bradford, England (n=47 583, 2010-19), Denmark (n=384 885, 2004-10), Finland (n=576 758, 2004-14), Norway (n=276 078, 2012-16) and Wales (n=844 478, 1986-2016). Number of cases/births for outcomes: stillbirth 2683/2 129 782, neonatal death 795/2 127 099, infant death 1676/2 127 099, low Apgar score at 5 minutes 31 633/1 780 158, NICU admission 1849/47 471. NICU=neonatal intensive care unit; error bars=95% confidence intervals

**Figure 4:**
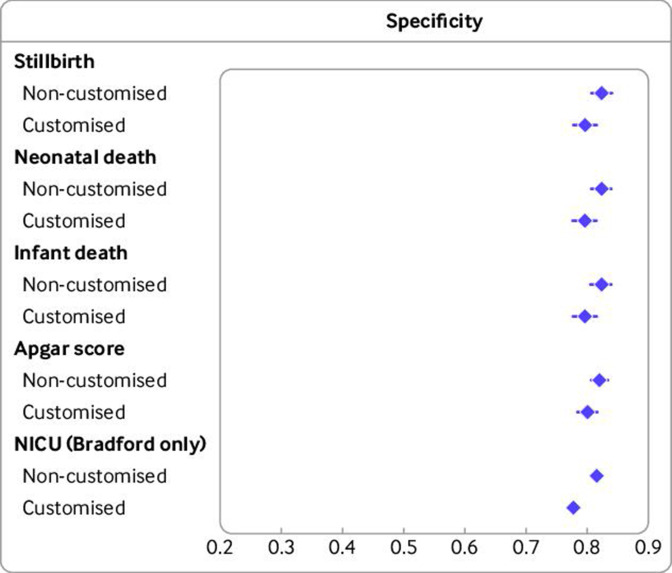
Pooled specificity of small-for-gestational-age (<10th age) and large-for-gestational-age (>90th centile) births versus average-for-gestational-age (10-90th centile) births for adverse perinatal outcomes with non-customised and customised birthweight centiles (n=2 129 782 births, 1986-2019). Pooled results are from Bradford, England (n=47 583, 2010-19), Denmark (n=384 885, 2004-10), Finland (n=576 758, 2004-14), Norway (n=276 078, 2012-16) and Wales (n=844 478, 1986-2016). Number of cases/births for outcomes: stillbirth 2683/2 129 782, neonatal death 795/2 127 099, infant death 1676/2 127 099, low Apgar score at 5 minutes 31 633/1 780 158, NICU admission 1849/47 471. NICU=neonatal intensive care unit; error bars=95% confidence intervals

Figures 3 and 4 (online supplemental table S4) show the pooled summary estimates of sensitivity and specificity, respectively, for combined SGA or LGA births versus AGA births, based on non-customised and customised centiles and (online supplemental table S5A-E show detailed country specific estimates). Results were similar for non-customised and customised charts, with confidence intervals overlapping. For example, for stillbirths, pooled sensitivity with non-customised and customised centiles was 0.34 (95% confidence interval 0.33 to 0.36) and 0.38 (0.36 to 0.40), respectively; and corresponding specificity was 0.82 (0.81 to 0.84) and 0.80 (0.78 to 0.82), respectively. Estimated sensitivity to the five outcomes ranged from 26% to 38%. Point estimates of sensitivity tended to be non-significantly higher for customised centiles for all mortality outcomes. Slightly higher specificity was observed for non-customised centiles than for customised centiles for all outcomes, particularly for NICU admission (based on Bradford data only).

Supplementary analyses exploring the sensitivity of the results from each dataset to their main sources of missing data (online supplemental tables S3A–E and S5A–E) showed highly similar results to the main analyses. Pooled results comparing the three Nordic datasets to the UK datasets were also similar with the exceptions of associations with neonatal and infant death (online supplemental figures S3 and S4). Associations of SGA births with these two outcomes were stronger in Nordic countries than in the UK for customised charts, while the associations of LGA births with the two outcomes were stronger in Nordic countries than UK for non-customised charts. However, results from these subgroup analyses were imprecise, with wide confidence intervals. Pooled results excluding Norway were similar to the main results (online supplemental figures S5 and S6). After comparing two different customisation methods to assign SGA/LGA values in Bradford data, we found that the results from customisation within studies were consistent with those from the bulk calculator, but the wide confidence intervals prevented making strong conclusions (online supplemental table S3A).

## Discussion

### Summary of main findings

In this comparative record linkage study, we did not find evidence that SGA or LGA births identified with customised birthweight centiles differed from non-customised centiles in their association with mortality (stillbirth, neonatal and infant mortality) or morbidity (low Apgar score and admission to NICU). However, the low sensitivity for both implies that a high proportion of infants at risk of these outcomes would not be identified by SGA or LGA with either chart.

### Study strengths and limitations

We used data from four nationally representative datasets of births (Denmark, Finland, Norway, and Wales) and one regional dataset (Bradford, England), enhancing statistical precision. The record linkage datasets were large and covered the whole population, so we were able to compare associations between non-customised and customised for rare outcomes such as perinatal mortality with greater precision than previous studies. The nature of our outcomes (mortality and admission to NICU) and the data from high income countries means very little, if any, outcome misclassification is likely. To our knowledge, this study is the largest so far of these associations; with over two million births, it is double the size of a previous systematic review^10^ and two previous record linkage studies of about one million births.^2 13^ However, we acknowledge that we still had limited precision for some estimates, and associations of LGA births with neonatal and perinatal deaths were particularly imprecise with wide confidence intervals. NICU admission data were also only available for Bradford data and customisation variables were missing for 33% of that data.

We restricted our analyses to term births to better distinguish fetal growth problems from preterm birth effects, and to facilitate comparisons with previous studies. However, we acknowledge that this restriction could introduce one form of selection bias (ie, healthier sample than those born prematurely) over another (we do not have weight for those continuing in utero, and centiles derived from babies born preterm do not reflect healthy fetal growth).^18^ The extent of missing data varied across different datasets; and for some dataset this was considerable (eg, data from Wales had missing weight for 54%). Nevertheless, results were similar in sensitivity analyses where we used either the restricted the data or partial customisation, and the customisation equation coefficients were similar across different datasets with different amounts of missing data (online supplemental table S2).

Here, we focused on the pooled analyses across all data sources but acknowledge some heterogeneity in estimates across locations. This heterogeneity could be due to differences in ethnic group (online supplemental tables S6-S10 detail the categories used in the different datasets) or other maternal characteristics, or could reflect differences between countries in fetal growth monitoring, how adverse perinatal outcomes are screened for, and their risk managed. The data come from different years within each country spanning from 1986 to 2019, during which time perinatal mortality has decreased globally.^19^ A change over time in the outcome is unlikely to affect associations of exposures with that outcome and neither would it affect the comparisons within countries of non-customised and customised SGA/LGA births with the outcomes we have explored here. However, differences within countries could exist in how strongly customised/non-customised centiles associate with the outcomes that are influenced by the different time periods that are covered in each study, for example, owing to differences and changes in the ethnic composition of the country. These differences within countries could bias our (pooled) analyses, but seems unlikely because we do not see differences between non-customised and customised SGA/LGA birth in their associations with outcomes within countries. In the future, if countries become, for example, more ethnically diverse, differences between countries in the strength of the association of customised, centile derived, SGA/LGA births could emerge and that might result in customised SGA/LGA births having stronger or weaker associations with adverse perinatal outcomes in different countries.

### Comparison with other studies

Consistent with our findings, a systematic review and meta-analysis did not find conclusive evidence of differences between SGA and LGA defined with customised and non-customised charts in predicting several important perinatal outcomes,^10^ and three subsequent studies of perinatal morbidity or mortality within countries have also shown no differences.^2 11 12^ In the previous meta-analysis,^10^ infants defined as SGA according to non-customised and customised charts had increased pooled risks compared with non-SGA infants for perinatal death (odds ratio 4.0 (95% confidence interval 2.8 to 5.1) *v* 5.8 (3.8 to 7.8)), neonatal death (2.9 (1.2 to 4.5) *v* 3.5 (1.1 to 8.0)), and NICU admission (2.4 (1.7 to 3.2) *v* 3.6 (2.0 to 5.5)). The authors concluded that the overlap of confidence intervals suggested little evidence of difference between the two charts but acknowledged that differences could become apparent with larger studies. With double the sample size of that previous study, we had considerably more precision to estimate associations with SGA births, but found that the confidence intervals for non-customised and customised estimates still overlapped. One consistency across all studies so far, including ours, is that associations of SGA births (defined by any of the charts) with adverse outcomes are stronger and more consistent than those associations of LGA births.

Two studies examined associations of SGA or LGA births with perinatal outcomes have compared customised centile charts to the Intergrowth-21st standards.^13 14^ Researchers from both studies concluded that the customised charts identified births at risk of being defined as SGA better than the Intergrowth-21st charts, and that the differences between the two charts were likely related to physiological differences by ethnic group. The larger study included 1.25 million births from 10 different countries, including Bhutan, China, India, the US, and six European countries, with the vast majority (97%) from the US or European countries.^13^ The results in relation to SGA births were driven by results from India (n=6436) and Bhutan (n=2779), which reflected <1% of the whole cohort.

We cannot directly compare our results to those from these previous studies because our study compared general population, non-customised reference charts rather than Intergrowth-21st charts. However, there could be some value in comparing these results to those from our study in the Bradford population (the most ethnically diverse of our data sources), where we found that SGA births based on non-customised charts had consistently higher relative risks for all outcomes than those based on customised charts, although with overlapping confidence intervals suggesting no statistical difference in associations. Based on these data, birthweight customisation did not appear to better identify a more at-risk group in Bradford, which had the highest proportions of infants with non-white maternal ethnicity and the lowest median birth weight compared with the other countries.

The role of ethnic group in customising fetal growth charts remains unclear, and its use in algorithms guiding clinical practice could normalise non-physiological differences that might even result in increasing ethnic inequalities in health.^20^ The customisation literature is unclear over how ethnic group should be conceptualised (self-identified ethnic group *v* country of origin/birth), which might also be important in relation to the extent to which ethnic group is appropriate to customise on. However, the promotion of customised charts is widespread globally and assumed to be measuring the same predictor. In all Nordic countries, parental country of birth/origin is often used as a measure of ethnic group, which is different to the concept of self-reported ethnic group (or race, for example, in the US) used in other countries. We feel strongly that in any research and development of global tools, such as customised centile charts, it is important to be clear about the meaning and justification of the measure of ethnic group.

### Implications of studies so far

The systematic review concluded that, given the limitations of observational studies, randomised controlled trials were needed to investigate the use of customised and non-customised charts in monitoring infant growth and in deciding when to intervene.^10^ However, given the low incidence of severe adverse outcomes such as stillbirth and neonatal mortality, alternative approaches including natural experiments^21^ or smaller adequately powered trials for surrogates^22^ are required to determine the efficacy of using customised versus non-customised charts in clinical settings. For example, a natural experiment compared change in stillbirth rates over time between Scotland, where very few units have adopted the growth assessment protocol (GAP, which uses customised charts), with the same in England; researchers concluded that little evidence existed to support GAP being more effective at reducing stillbirth than standard care.^21^

In the first large randomised controlled trial to compare GAP with standard care, including over 180 000 deliveries from 13 obstetric units in the UK, researchers found no difference between GAP and standard care for the primary outcome of antenatal detection of SGA births, the proposed route through which GAP is hypothesised to reduce stillbirth.^23^ Evidence of a lower stillbirth rate was seen—which could have been a chance finding, given that researchers saw no differences in the other 24 secondary outcomes and no effect on the primary outcome that would be the mechanism for reducing stillbirth.^23^ The natural experiment and randomised controlled trials did not test whether customised charts were better than non-customised charts at identifying pregnancies at risk, and results for both chart types will be influenced by the effects of standard care in the comparison group. Thus, large observational studies remain the most feasible design for answering questions about which centile chart best predicts adverse perinatal outcomes.

Our results and those of previous studies suggest that SGA and LGA births that are defined by the bottom and top deciles of birthweight distribution in general have poor general predictive ability for these rare outcomes, irrespective of using non-customised or customised charts. We found similar high specificity and low sensitivity for non-customised and customised charts. Taking the pooled point estimate of stillbirths as an example, our findings suggest that non-customised and customised SGA/LGA births could identify, on average, 34% and 38%, respectively, of those who went on to have a stillbirth, missing more than 60% of events. Recent literature on birthweight centiles shows that no cut-off point for SGA performs well for predicting neonatal morbidity and mortality.^24–27^ Our aim was not to develop models to predict outcomes on an individual basis, but to assess whether customised centiles of birth weight were more strongly associated with outcomes than non-customised centiles, at the population level. On an individual level, continuous birth weight combined with multivariable predictive models could have improved predictive accuracy. Our analyses also did not assess wider programmes of plotting fetal growth and the benefits of reacting to changes in expected growth rates, such as faltering fundal height measurements, which remains an important area of research. Nevertheless, the use of customised charts in clinical practice requires more resources, so their clinical benefit would need to be demonstrable and balanced with their cost.

## Data Availability

Data may be obtained from a third party and are not publicly available. The register data can be used for scientific purposes by approved researchers by application to the relevant data-holding authorities of each country. For statistical code, please contact fanny.kilpi@bristol.ac.uk.
