# Supplementary material for: Association between perinatal mortality and morbidity and customised and non-customised birthweight centiles in Denmark, Finland, Norway, Wales, and England: comparative, population based, record linkage study
Source: BMJ Med. 2023 Aug 30;2(1):e000521. doi: 10.1136/bmjmed-2023-000521 (PMC10471867; doi:10.1136/bmjmed-2023-000521)
Supplement: Supplementary data [file bmjmed-2023-000521supp001.pdf]

SUPPLEMENTARY MATERIAL

Supplementary methods

Birthweight centile customisation

Customisation was proposed by the group who lead the Perinatal Institute, with many previous studies using the Perinatal Institute methods and calculators to generate customised centiles. In Bradford data, we used the Perinatal Institute's global bulk calculator (version 8.0.4)[1] for deriving customised birthweight centiles. The calculator requires hosting participant data on the Institute's server whilst doing these calculations, and therefore, for information governance reasons, we could not use the calculator for customisation in other country data sets. The Perinatal Institute does not provide exact details of the coefficients used in their customization calculator, therefore we developed customised centiles within each dataset following the approach used by the Perinatal Institute as closely as possible from information on their website.[2] First, birthweight was regressed on gestational age, sex, maternal weight, height, parity, and ethnicity in observations with complete data on customisation variables. In the model, gestational age (in days) was centred at 280, and height and weight were centred at the population median, with quadratic and cubic terms added for weight. Parity was included as dummy variables (0 [reference category]/ 1 previous birth/ 2 previous births/ 3 previous births/ 4 or more previous births), and sex coded -1 for male infant and 1 for female. Dummy variables were included for all available ethnicity groups (with the majority group as the reference group).

We saved the coefficients, the constant and residual standard error (SE) from the regression (details in Table S4). The constant and the residual SE were used to derive the coefficient of variation (CV) (residual SE*100/constant). This was used to calculate the 10th and 90th centile limits by multiplying the CV by the relevant z-score (i.e. -1.282 and 1.282, respectively).

For each observation (regardless of whether there was complete data on variables), we predicted birthweight at 280 days (term) based on the coefficients and the available information on infant sex and maternal height, weight, parity, and ethnic group. In the main analyses, we used all available data, assigning missing values with population median for height and weight and reference values for parity and ethnicity. In other words, when a birth observation had missing data on a customisation covariate such as weight, this coefficient would in effect drop from the equation for that observation, because the value would be 0 (the centred median value).

We used the same equation as the Perinatal Institute (for gestational weeks ≥25) to derive the percentage birthweight expected for the specific gestational age compared to that at 280 days (term). Based on this, we calculated the "optimal" birthweight for the specific gestational age as a percentage of the term birthweight. Using the centile limits derived with the CV, we calculated whether the actual observed birthweight was above or below the 10th (SGA) and 90th (LGA) centiles of the optimal birthweight expected for an infant born at the same gestational age, with the same sex and maternal characteristics.

Data sets and sensitivity analyses

We conducted sensitivity analyses to assess the effect of the patterns of missing data on customisation variables in different countries, as described below.

Kilpi F, *et al. BMJMED* 2023; 2:e000521. doi: 10.1136/bmjmed-2023-000521

BRADFORD

Bradford obstetric data was available for the years 2010-2019. In the main analysis, we performed full customisation using all available data. Missing ethnicity, or ethnicity categories that did not conform to those available in the Perinatal global bulk calculator (i.e. Any other Asian, Any other Black, Any other White, Any other ethnic group, Any other mixed background, Not stated, White and Asian, White and Black African, White and Black Caribbean) (Table S6), were assigned the "global average" option (26%) as suggested by the Perinatal Institute. In sensitivity analyses, we restricted the dataset to observations with maternal ethnicity that we were able to categorise. In a further sensitivity analysis using the latter restricted dataset, we calculated within-study customised SGA/LGA using the customization equations as for the other datasets.

DENMARK

Danish birth registry data was available for the whole population from 1980 to 2010, but maternal weight and height only became available from 2004. The main analysis is therefore based on data from 2004-2010. Maternal ethnicity was based on maternal country of origin, linked from the population register. In our main analysis, we performed full customisation in the data for years 2004-2010. In a sensitivity analysis, we performed partial customisation using only ethnicity and parity in data from the years 1980-2010.

FINLAND

Finnish data derives from birth registry of all births from the year 2004, when maternal height and weight data became available, up to the year 2014. Maternal ethnicity is based on mother's country of birth linked from the population register.

NORWAY

Norwegian data derived from the birth registry from years 1999 to 2016. Exclusion by major congenital anomalies was not possible. Maternal ethnicity is based on mother's country of birth, linked from the population register. The main analysis is restricted to the years 2012 to 2016 when weight and height are available for full customisation, but partial customisation with only ethnicity and parity was performed for the data in years 1999 to 2016.

WALES

Welsh data on births from 1987 to 2016 was available through the SAIL databank.[3,4] The linkage combined data from the National Community Child Health Database, the Annual District Birth Extract, the Annual District Death Extract, CARIS and GP records. Maternal height and weight were derived from the most recent measurement prior to pregnancy from GP records (median years before pregnancy 1.5, IQR 1-5, and 1.7, IQR 0.7 to 3.6, respectively). Ethnicity was categorised using the post-31st March 2002 keys from NHS Wales Data Dictionary (see Table S7).

In the main analysis, we used full customisation in the full data set (years 1987-2016). Prior to 2003, more than 50% of ethnicity data was missing, and prior to 1999-2001, more than 50% of data on height and weight were missing. Therefore, we also performed a sensitivity analysis using full customisation in data restricted to years 2003-2016.

References

1. Gardosi J, Williams A, Hugh O, Francis A. Customised Centile Calculator. GROW version 8.0.4. Gestation Network;

2. GROW documentation [Internet]. [cited 2021 Apr 29]. Available from: https://www.gestation.net/GROW_documentation.pdf

3. Lyons RA, Jones KH, John G, Brooks CJ, Verplancke JP, Ford D V., et al. The SAIL databank: Linking multiple health and social care datasets. BMC Med Inform Decis Mak. BioMed Central Ltd.; 2009;9.

4. Ford D V., Jones KH, Verplancke JP, Lyons RA, John G, Brown G, et al. The SAIL Databank: Building a national architecture for e-health research and evaluation. BMC Health Serv Res. BMC Health Serv Res; 2009;9.

BMJ Publishing Group Limited (BMJ) disclaims all liability and responsibility arising from any reliance placed on this supplemental material which has been supplied by the author(s)

FIGURE S1. Meta-analysis of risk ratios of perinatal adverse outcomes by SGA vs AGA (<10th vs 10-90th) with non-customised and customised birthweight centiles (Bradford N=47,583, 2010-2019; Denmark N=384,885, 2004-2010; Finland N=576,758, 2004-2014; Norway N=276,078, 2012-2016; Wales N=844,478, 1986-2016)

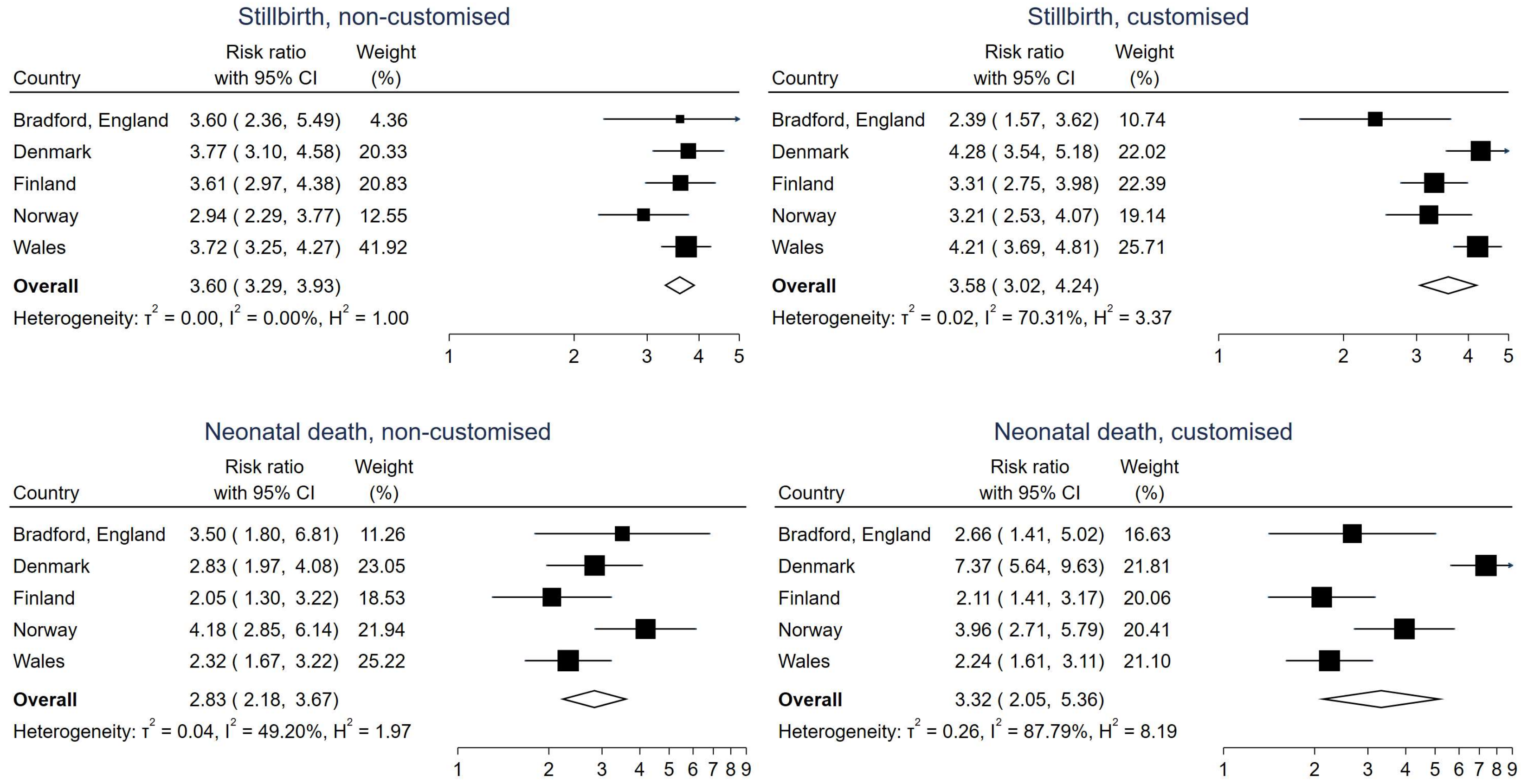

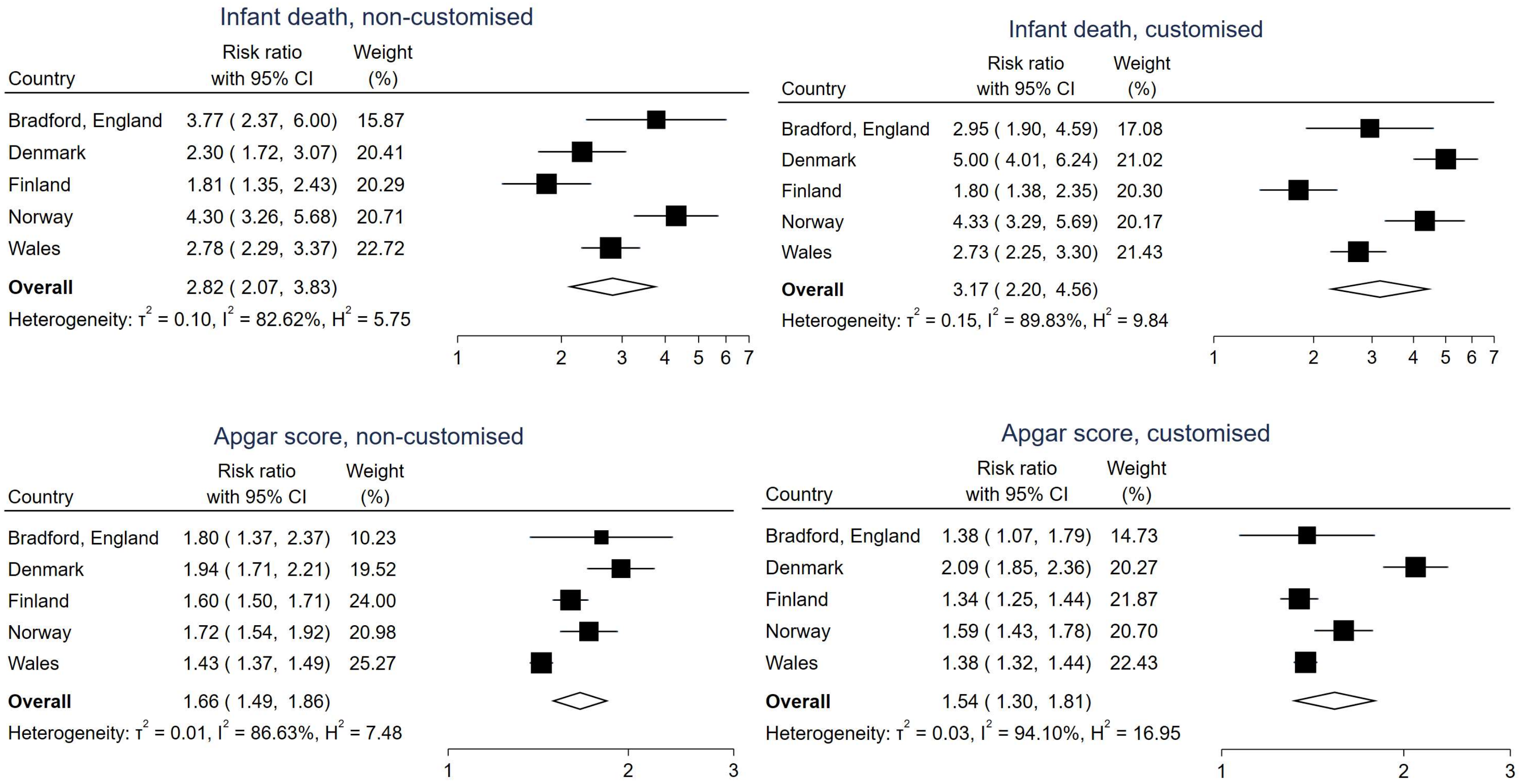
Infant death, non-customised
Country | Risk ratio with 95% CI | Weight (%)
Bradford, England 3.77 (2.37, 6.00) 15.87
Denmark 2.30 (1.72, 3.07) 20.41
Finland 1.81 (1.35, 2.43) 20.29
Norway 4.30 (3.26, 5.68) 20.71
Wales 2.78 (2.29, 3.37) 22.72
Overall 2.82 (2.07, 3.83)
Heterogeneity: τ² = 0.10, I² = 82.62%, H² = 5.75
1 2 3 4 5 6 7
Infant death, customised
Country | Risk ratio with 95% CI | Weight (%)
Bradford, England 2.95 (1.90, 4.59) 17.08
Denmark 5.00 (4.01, 6.24) 21.02
Finland 1.80 (1.38, 2.35) 20.30
Norway 4.33 (3.29, 5.69) 20.17
Wales 2.73 (2.25, 3.30) 21.43
Overall 3.17 (2.20, 4.56)
Heterogeneity: τ² = 0.15, I² = 89.83%, H² = 9.84
1 2 3 4 5 6 7
Apgar score, non-customised
Country | Risk ratio with 95% CI | Weight (%)
Bradford, England 1.80 (1.37, 2.37) 10.23
Denmark 1.94 (1.71, 2.21) 19.52
Finland 1.60 (1.50, 1.71) 24.00
Norway 1.72 (1.54, 1.92) 20.98
Wales 1.43 (1.37, 1.49) 25.27
Overall 1.66 (1.49, 1.86)
Heterogeneity: τ² = 0.01, I² = 86.63%, H² = 7.48
1 2 3
Apgar score, customised
Country | Risk ratio with 95% CI | Weight (%)
Bradford, England 1.38 (1.07, 1.79) 14.73
Denmark 2.09 (1.85, 2.36) 20.27
Finland 1.34 (1.25, 1.44) 21.87
Norway 1.59 (1.43, 1.78) 20.70
Wales 1.38 (1.32, 1.44) 22.43
Overall 1.54 (1.30, 1.81)
Heterogeneity: τ² = 0.03, I² = 94.10%, H² = 16.95
1 2 3

FIGURE S2. Meta-analysis of risk ratios of perinatal adverse outcomes by LGA vs AGA (>90th vs 10-90th) with non-customised and customised birthweight centiles (Bradford N=47,583, 2010-2019; Denmark N=384,885, 2004-2010; Finland N=576,758, 2004-2014; Norway N=276,078, 2012-2016; Wales N=844,478, 1986-2016)

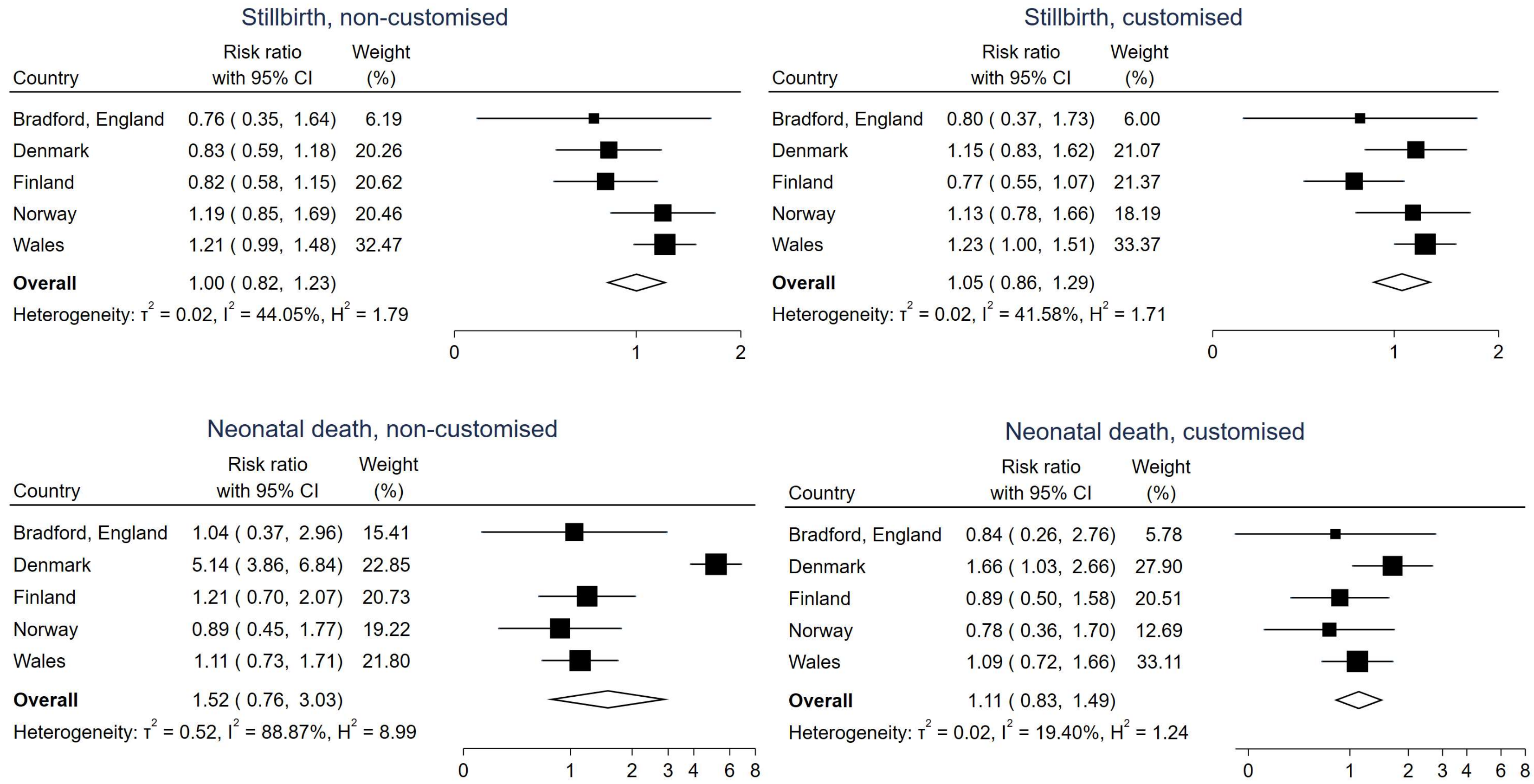

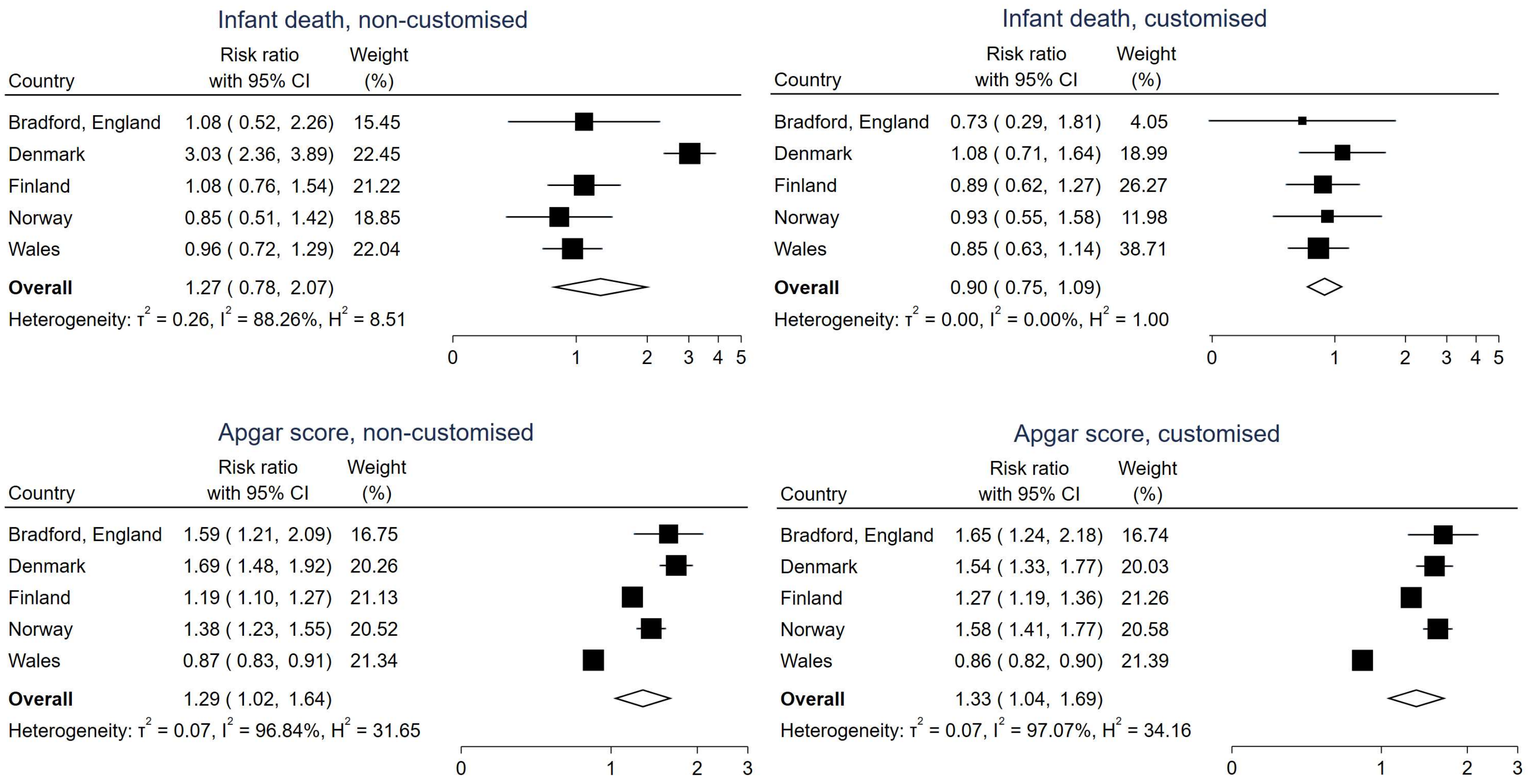
Infant death, non-customised
Country
Risk ratio with 95% CI
Weight (%)
Bradford, England 1.08 (0.52, 2.26) 15.45
Denmark 3.03 (2.36, 3.89) 22.45
Finland 1.08 (0.76, 1.54) 21.22
Norway 0.85 (0.51, 1.42) 18.85
Wales 0.96 (0.72, 1.29) 22.04
Overall 1.27 (0.78, 2.07)
Heterogeneity: τ² = 0.26, I² = 88.26%, H² = 8.51
0 1 2 3 4 5
Infant death, customised
Country
Risk ratio with 95% CI
Weight (%)
Bradford, England 0.73 (0.29, 1.81) 4.05
Denmark 1.08 (0.71, 1.64) 18.99
Finland 0.89 (0.62, 1.27) 26.27
Norway 0.93 (0.55, 1.58) 11.98
Wales 0.85 (0.63, 1.14) 38.71
Overall 0.90 (0.75, 1.09)
Heterogeneity: τ² = 0.00, I² = 0.00%, H² = 1.00
0 1 2 3 4 5
Apgar score, non-customised
Country
Risk ratio with 95% CI
Weight (%)
Bradford, England 1.59 (1.21, 2.09) 16.75
Denmark 1.69 (1.48, 1.92) 20.26
Finland 1.19 (1.10, 1.27) 21.13
Norway 1.38 (1.23, 1.55) 20.52
Wales 0.87 (0.83, 0.91) 21.34
Overall 1.29 (1.02, 1.64)
Heterogeneity: τ² = 0.07, I² = 96.84%, H² = 31.65
0 1 2 3
Apgar score, customised
Country
Risk ratio with 95% CI
Weight (%)
Bradford, England 1.65 (1.24, 2.18) 16.74
Denmark 1.54 (1.33, 1.77) 20.03
Finland 1.27 (1.19, 1.36) 21.26
Norway 1.58 (1.41, 1.77) 20.58
Wales 0.86 (0.82, 0.90) 21.39
Overall 1.33 (1.04, 1.69)
Heterogeneity: τ² = 0.07, I² = 97.07%, H² = 34.16
0 1 2 3

FIGURE S3 – Meta-analysis of risk ratios of perinatal adverse outcomes by SGA vs AGA (<10th vs 10-90th) with non-customised and customised birthweight centiles by country sub-groups (Bradford N=47,583, 2010-2019; Denmark N=384,885, 2004-2010; Finland N=576,758, 2004-2014; Norway N=276,078, 2012-2016; Wales N=844,478, 1986-2016)

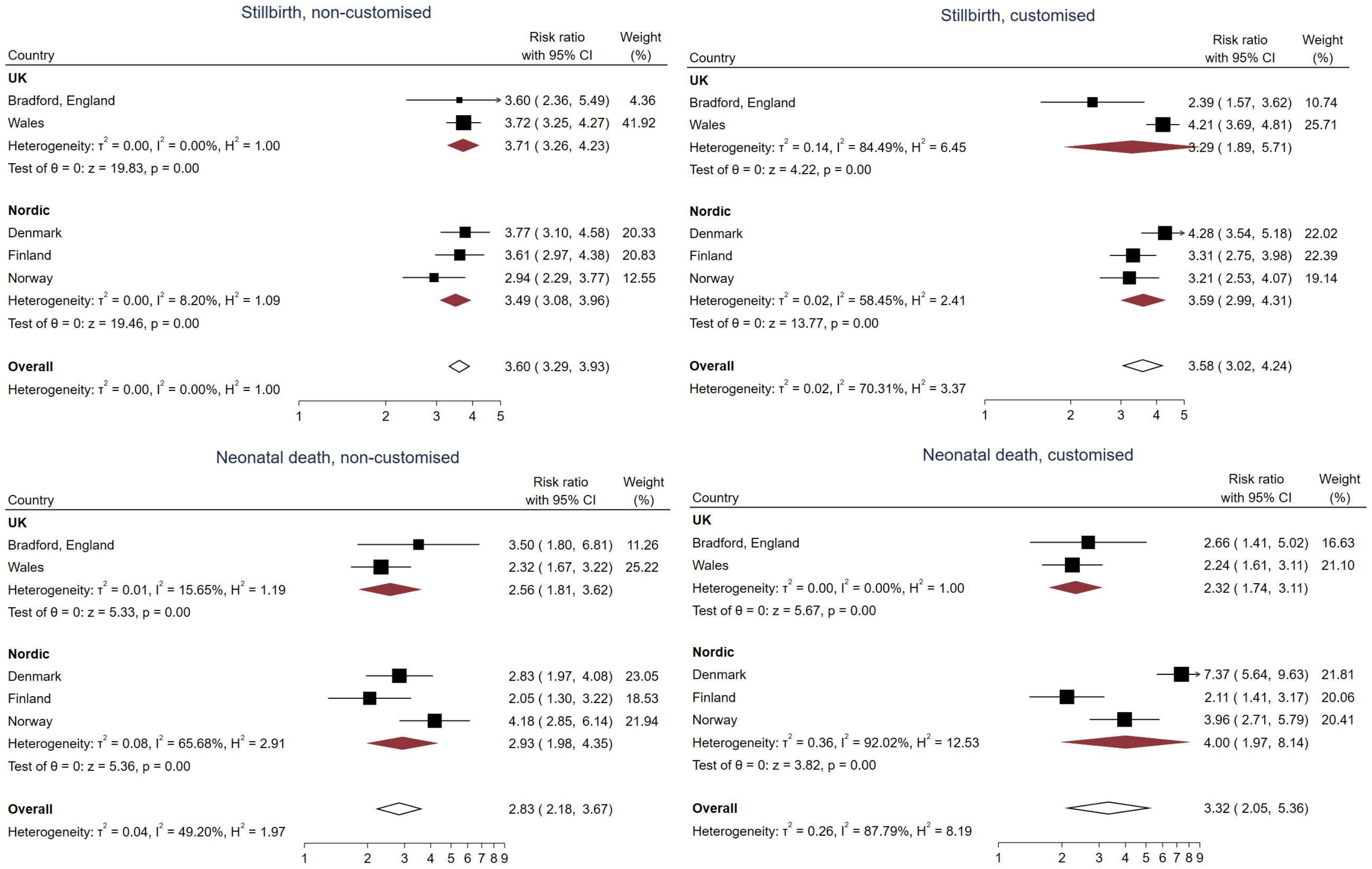

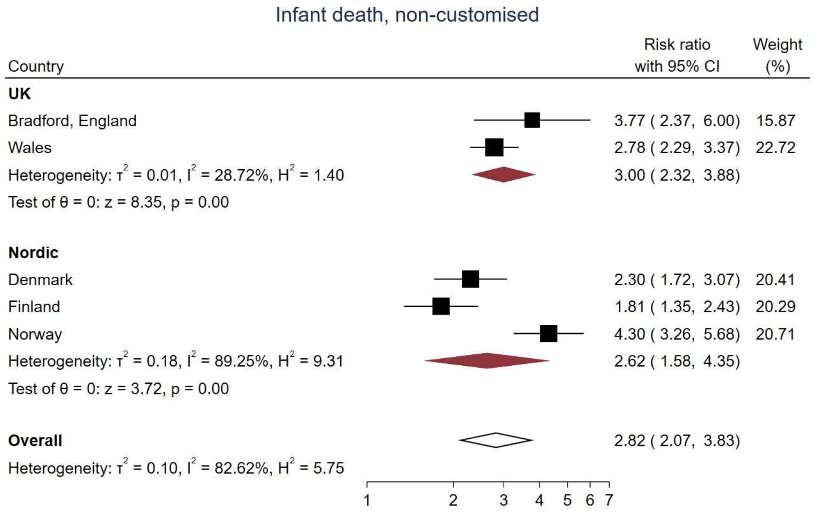
Infant death, non-customised
Country
Risk ratio with 95% CI
Weight (%)
UK
Bradford, England 3.77 (2.37, 6.00) 15.87
Wales 2.78 (2.29, 3.37) 22.72
Heterogeneity: τ^2 = 0.01, I^2 = 28.72%, H^2 = 1.40 3.00 (2.32, 3.88)
Test of θ = 0: z = 8.35, p = 0.00
Nordic
Denmark 2.30 (1.72, 3.07) 20.41
Finland 1.81 (1.35, 2.43) 20.29
Norway 4.30 (3.26, 5.68) 20.71
Heterogeneity: τ^2 = 0.18, I^2 = 89.25%, H^2 = 9.31 2.62 (1.58, 4.35)
Test of θ = 0: z = 3.72, p = 0.00
Overall 2.82 (2.07, 3.83)
Heterogeneity: τ^2 = 0.10, I^2 = 82.62%, H^2 = 5.75
1 2 3 4 5 6 7

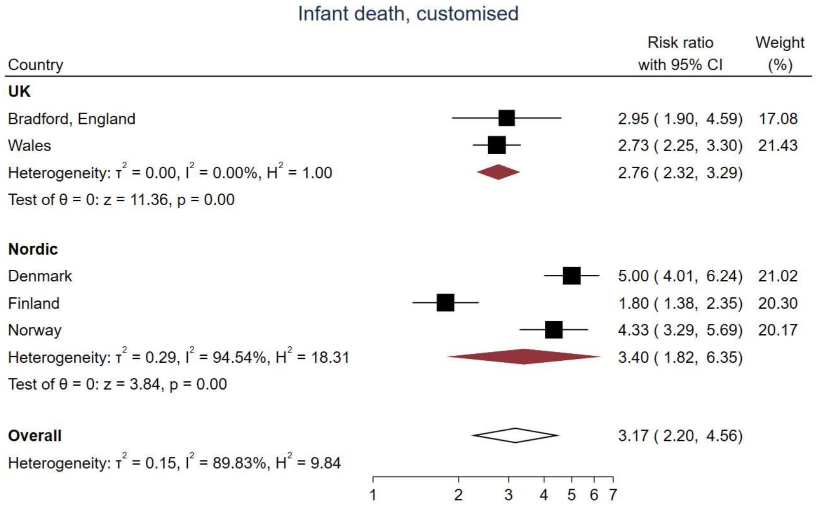
Infant death, customised
Country
Risk ratio with 95% CI
Weight (%)
UK
Bradford, England 2.95 (1.90, 4.59) 17.08
Wales 2.73 (2.25, 3.30) 21.43
Heterogeneity: τ^2 = 0.00, I^2 = 0.00%, H^2 = 1.00 2.76 (2.32, 3.29)
Test of θ = 0: z = 11.36, p = 0.00
Nordic
Denmark 5.00 (4.01, 6.24) 21.02
Finland 1.80 (1.38, 2.35) 20.30
Norway 4.33 (3.29, 5.69) 20.17
Heterogeneity: τ^2 = 0.29, I^2 = 94.54%, H^2 = 18.31 3.40 (1.82, 6.35)
Test of θ = 0: z = 3.84, p = 0.00
Overall 3.17 (2.20, 4.56)
Heterogeneity: τ^2 = 0.15, I^2 = 89.83%, H^2 = 9.84
1 2 3 4 5 6 7

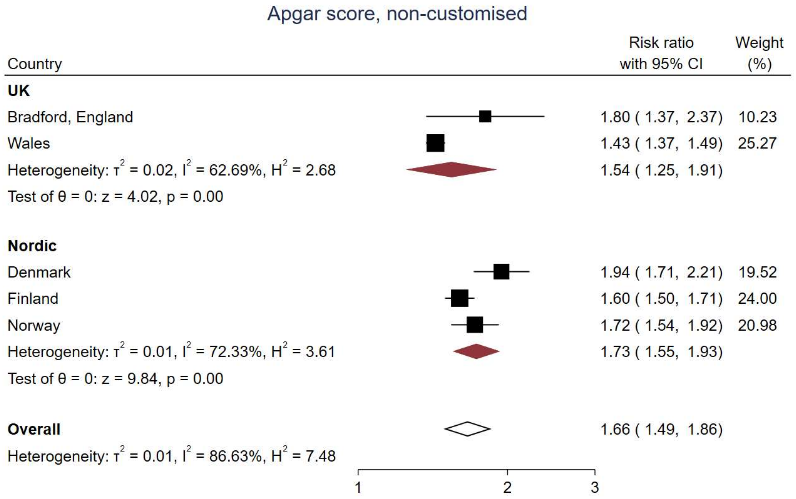
Apgar score, non-customised
Country
Risk ratio with 95% CI
Weight (%)
UK
Bradford, England 1.80 (1.37, 2.37) 10.23
Wales 1.43 (1.37, 1.49) 25.27
Heterogeneity: τ^2 = 0.02, I^2 = 62.69%, H^2 = 2.68 1.54 (1.25, 1.91)
Test of θ = 0: z = 4.02, p = 0.00
Nordic
Denmark 1.94 (1.71, 2.21) 19.52
Finland 1.60 (1.50, 1.71) 24.00
Norway 1.72 (1.54, 1.92) 20.98
Heterogeneity: τ^2 = 0.01, I^2 = 72.33%, H^2 = 3.61 1.73 (1.55, 1.93)
Test of θ = 0: z = 9.84, p = 0.00
Overall 1.66 (1.49, 1.86)
Heterogeneity: τ^2 = 0.01, I^2 = 86.63%, H^2 = 7.48
1 2 3

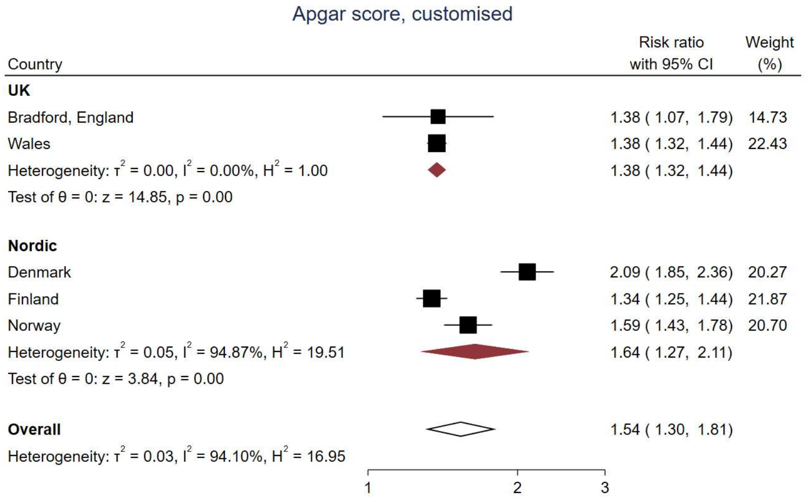
Apgar score, customised
Country
Risk ratio with 95% CI
Weight (%)
UK
Bradford, England 1.38 (1.07, 1.79) 14.73
Wales 1.38 (1.32, 1.44) 22.43
Heterogeneity: τ^2 = 0.00, I^2 = 0.00%, H^2 = 1.00 1.38 (1.32, 1.44)
Test of θ = 0: z = 14.85, p = 0.00
Nordic
Denmark 2.09 (1.85, 2.36) 20.27
Finland 1.34 (1.25, 1.44) 21.87
Norway 1.59 (1.43, 1.78) 20.70
Heterogeneity: τ^2 = 0.05, I^2 = 94.87%, H^2 = 19.51 1.64 (1.27, 2.11)
Test of θ = 0: z = 3.84, p = 0.00
Overall 1.54 (1.30, 1.81)
Heterogeneity: τ^2 = 0.03, I^2 = 94.10%, H^2 = 16.95
1 2 3

FIGURE S4 – Meta-analysis of risk ratios of perinatal adverse outcomes by LGA (>90th vs 10-90th) with non-customised and customised birthweight centiles by country sub-groups (Bradford N=47,583, 2010-2019; Denmark N=384,885, 2004-2010; Finland N=576,758, 2004-2014; Norway N=276,078, 2012-2016; Wales N=844,478, 1986-2016)

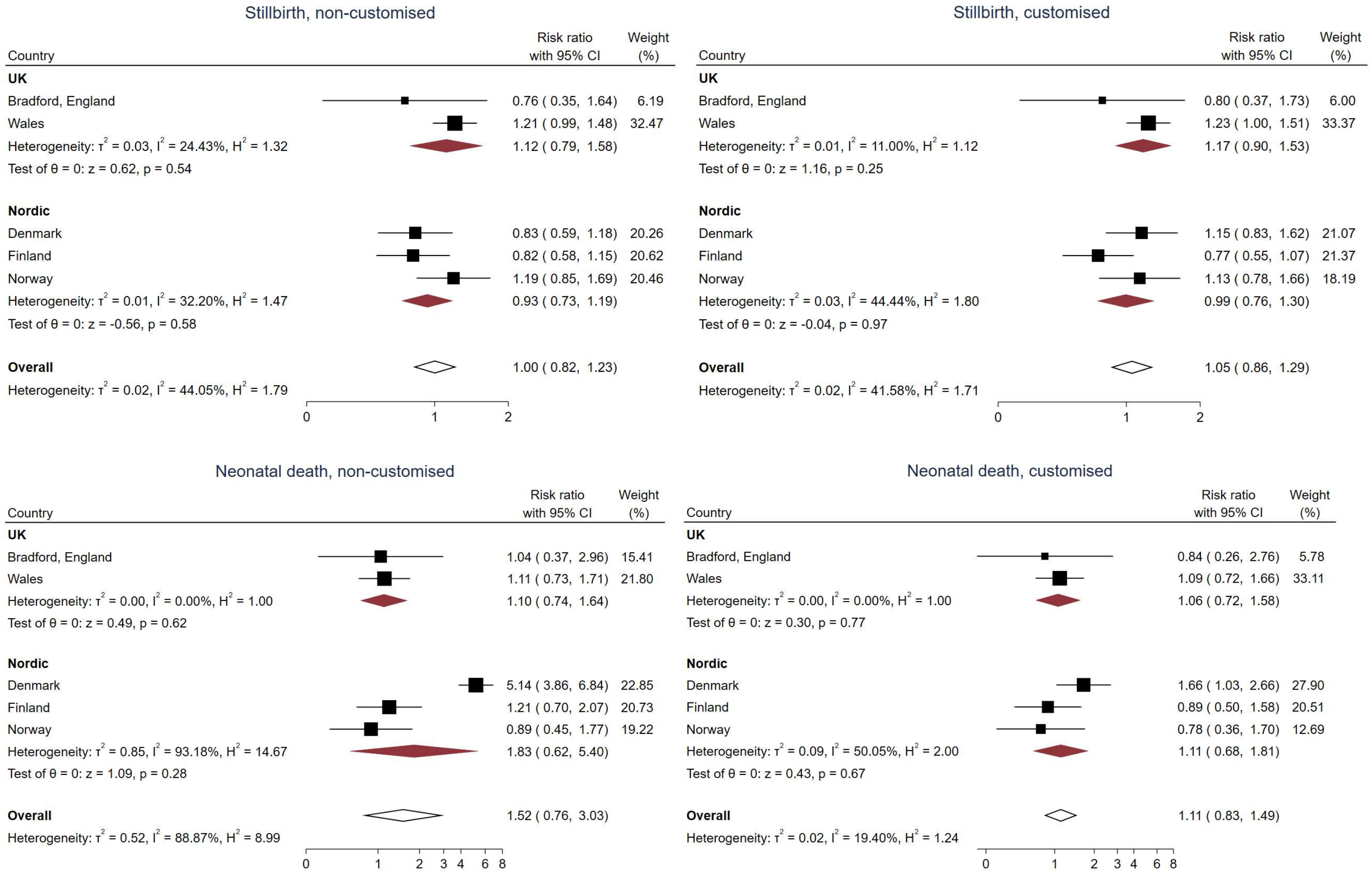

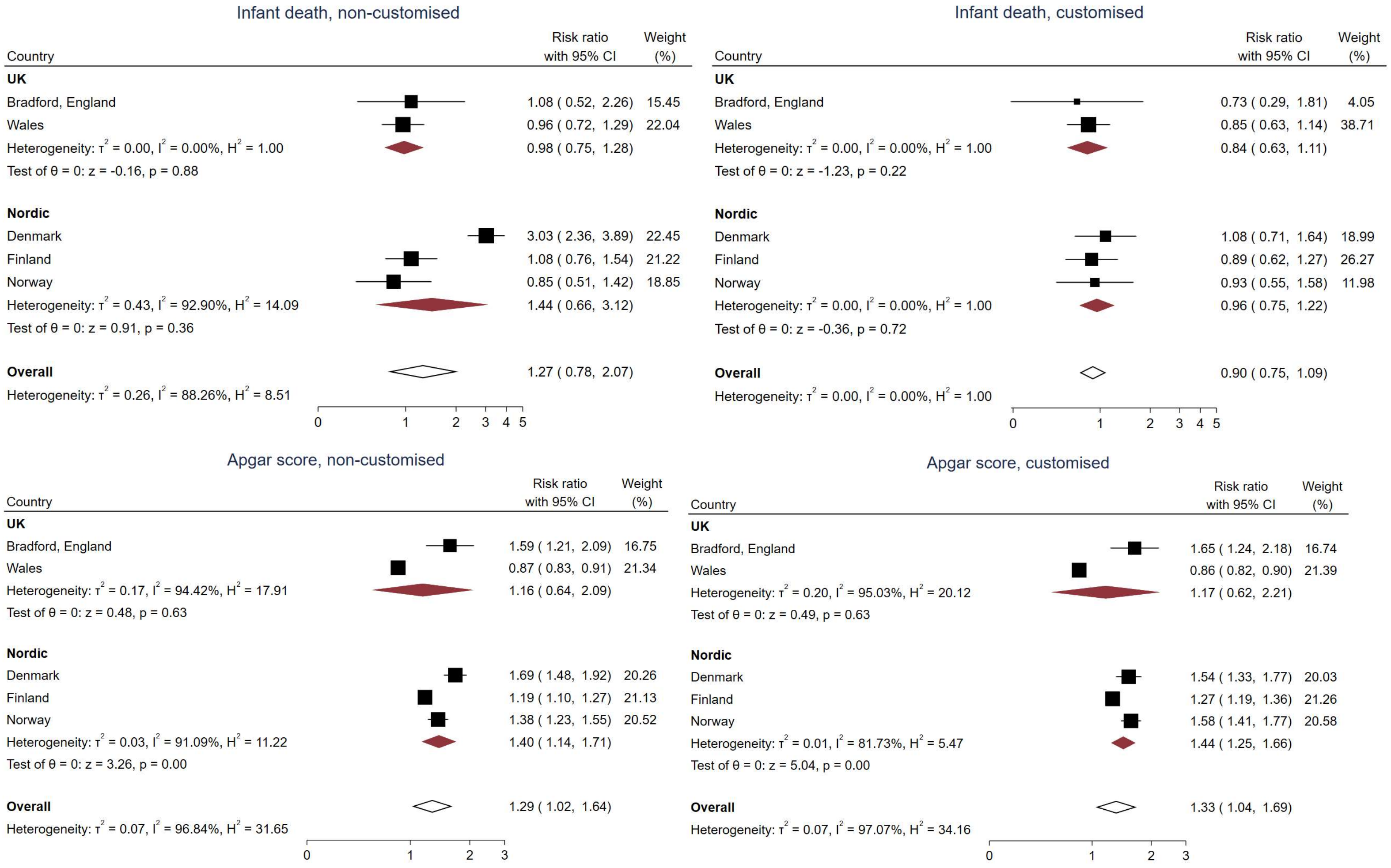
Infant death, non-customised
Country | Risk ratio with 95% CI | Weight (%)
UK
Bradford, England 1.08 (0.52, 2.26) 15.45
Wales 0.96 (0.72, 1.29) 22.04
Heterogeneity: τ² = 0.00, I² = 0.00%, H² = 1.00 0.98 (0.75, 1.28)
Test of θ = 0: z = -0.16, p = 0.88
Nordic
Denmark 3.03 (2.36, 3.89) 22.45
Finland 1.08 (0.76, 1.54) 21.22
Norway 0.85 (0.51, 1.42) 18.85
Heterogeneity: τ² = 0.43, I² = 92.90%, H² = 14.09 1.44 (0.66, 3.12)
Test of θ = 0: z = 0.91, p = 0.36
Overall 1.27 (0.78, 2.07)
Heterogeneity: τ² = 0.26, I² = 88.26%, H² = 8.51
0 1 2 3 4 5
Infant death, customised
Country | Risk ratio with 95% CI | Weight (%)
UK
Bradford, England 0.73 (0.29, 1.81) 4.05
Wales 0.85 (0.63, 1.14) 38.71
Heterogeneity: τ² = 0.00, I² = 0.00%, H² = 1.00 0.84 (0.63, 1.11)
Test of θ = 0: z = -1.23, p = 0.22
Nordic
Denmark 1.08 (0.71, 1.64) 18.99
Finland 0.89 (0.62, 1.27) 26.27
Norway 0.93 (0.55, 1.58) 11.98
Heterogeneity: τ² = 0.00, I² = 0.00%, H² = 1.00 0.96 (0.75, 1.22)
Test of θ = 0: z = -0.36, p = 0.72
Overall 0.90 (0.75, 1.09)
Heterogeneity: τ² = 0.00, I² = 0.00%, H² = 1.00
0 1 2 3 4 5
Apgar score, non-customised
Country | Risk ratio with 95% CI | Weight (%)
UK
Bradford, England 1.59 (1.21, 2.09) 16.75
Wales 0.87 (0.83, 0.91) 21.34
Heterogeneity: τ² = 0.17, I² = 94.42%, H² = 17.91 1.16 (0.64, 2.09)
Test of θ = 0: z = 0.48, p = 0.63
Nordic
Denmark 1.69 (1.48, 1.92) 20.26
Finland 1.19 (1.10, 1.27) 21.13
Norway 1.38 (1.23, 1.55) 20.52
Heterogeneity: τ² = 0.03, I² = 91.09%, H² = 11.22 1.40 (1.14, 1.71)
Test of θ = 0: z = 3.26, p = 0.00
Overall 1.29 (1.02, 1.64)
Heterogeneity: τ² = 0.07, I² = 96.84%, H² = 31.65
0 1 2 3
Apgar score, customised
Country | Risk ratio with 95% CI | Weight (%)
UK
Bradford, England 1.65 (1.24, 2.18) 16.74
Wales 0.86 (0.82, 0.90) 21.39
Heterogeneity: τ² = 0.20, I² = 95.03%, H² = 20.12 1.17 (0.62, 2.21)
Test of θ = 0: z = 0.49, p = 0.63
Nordic
Denmark 1.54 (1.33, 1.77) 20.03
Finland 1.27 (1.19, 1.36) 21.26
Norway 1.58 (1.41, 1.77) 20.58
Heterogeneity: τ² = 0.01, I² = 81.73%, H² = 5.47 1.44 (1.25, 1.66)
Test of θ = 0: z = 5.04, p = 0.00
Overall 1.33 (1.04, 1.69)
Heterogeneity: τ² = 0.07, I² = 97.07%, H² = 34.16
0 1 2 3

FIGURE S5 – Meta-analysis of risk ratios of perinatal adverse outcomes by SGA vs AGA (<10th vs 10-90th) with non-customised and customised birthweight centiles excluding Norway (Bradford N=47,583, 2010-2019; Denmark N=384,885, 2004-2010; Finland N=576,758, 2004-2014; Wales N=844,478, 1986-2016)

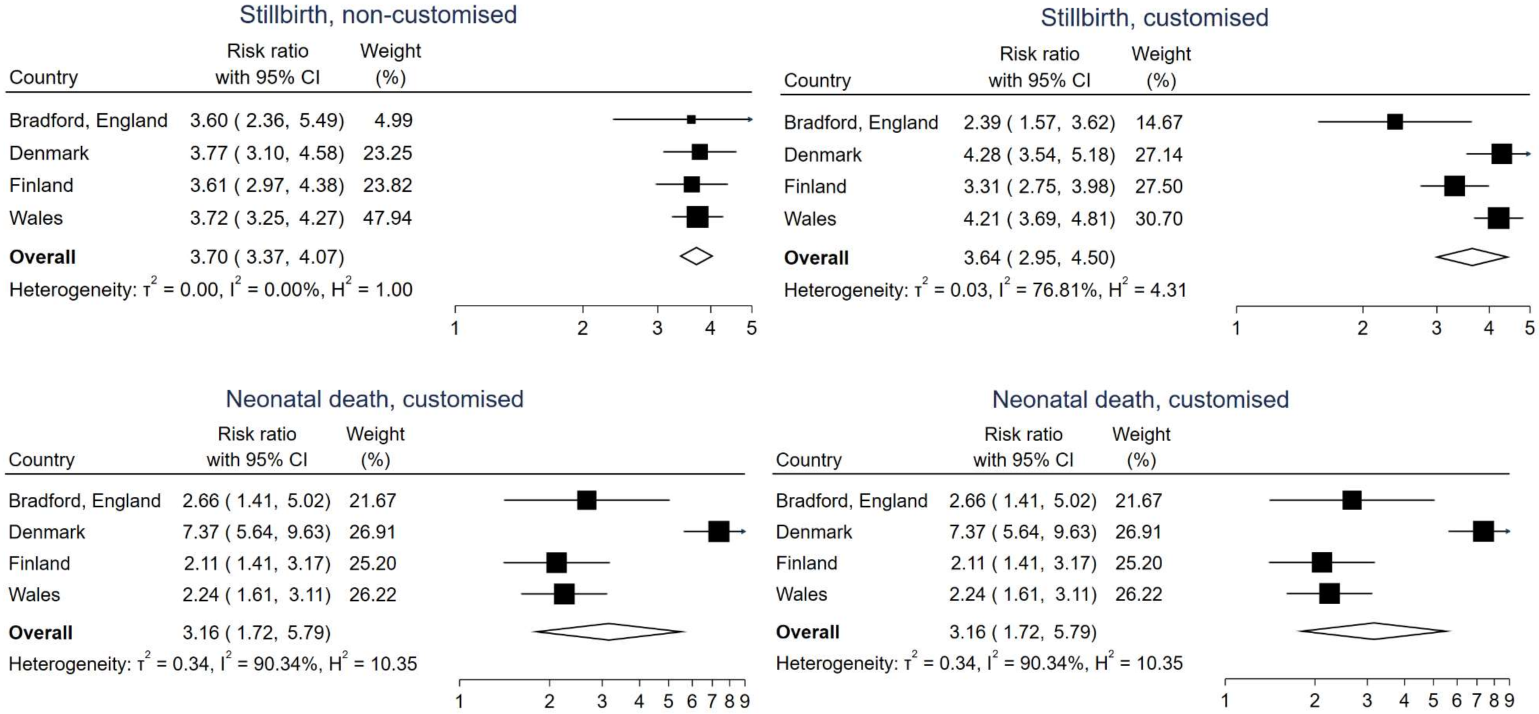

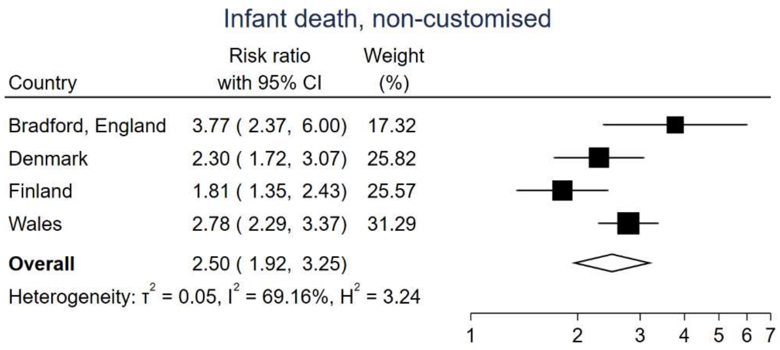

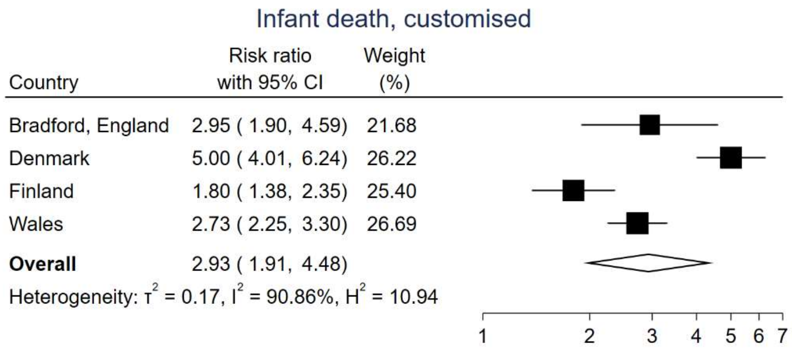

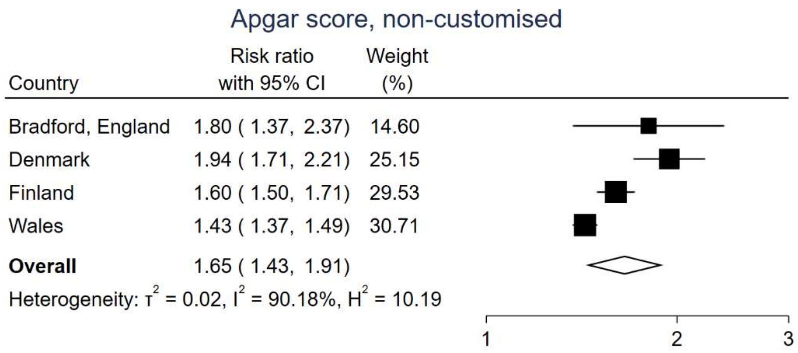

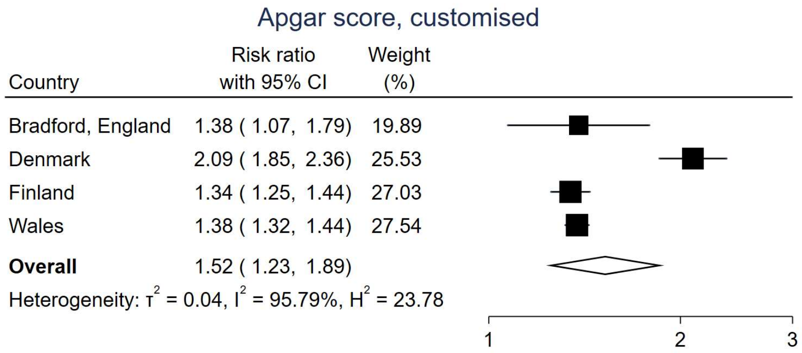

Kilpi F, *et al. BMJMED* 2023; 2:e000521. doi: 10.1136/bmjmed-2023-000521

FIGURE S6 – Meta-analysis of risk ratios of perinatal adverse outcomes by LGA (>90th vs 10-90th) with non-customised and customised birthweight centiles excluding Norway (Bradford N=47,583, 2010-2019; Denmark N=384,885, 2004-2010; Finland N=576,758, 2004-2014; Wales N=844,478, 1986-2016)

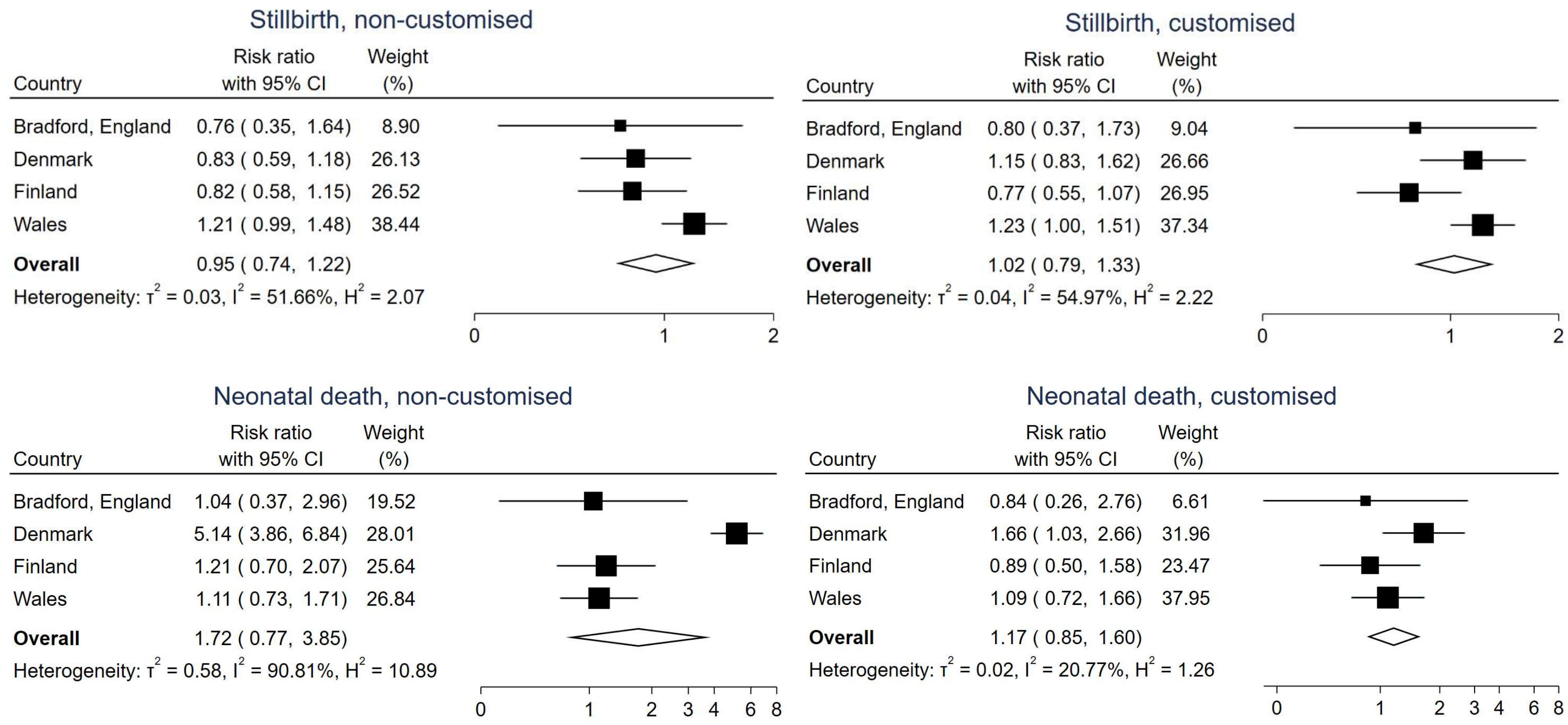

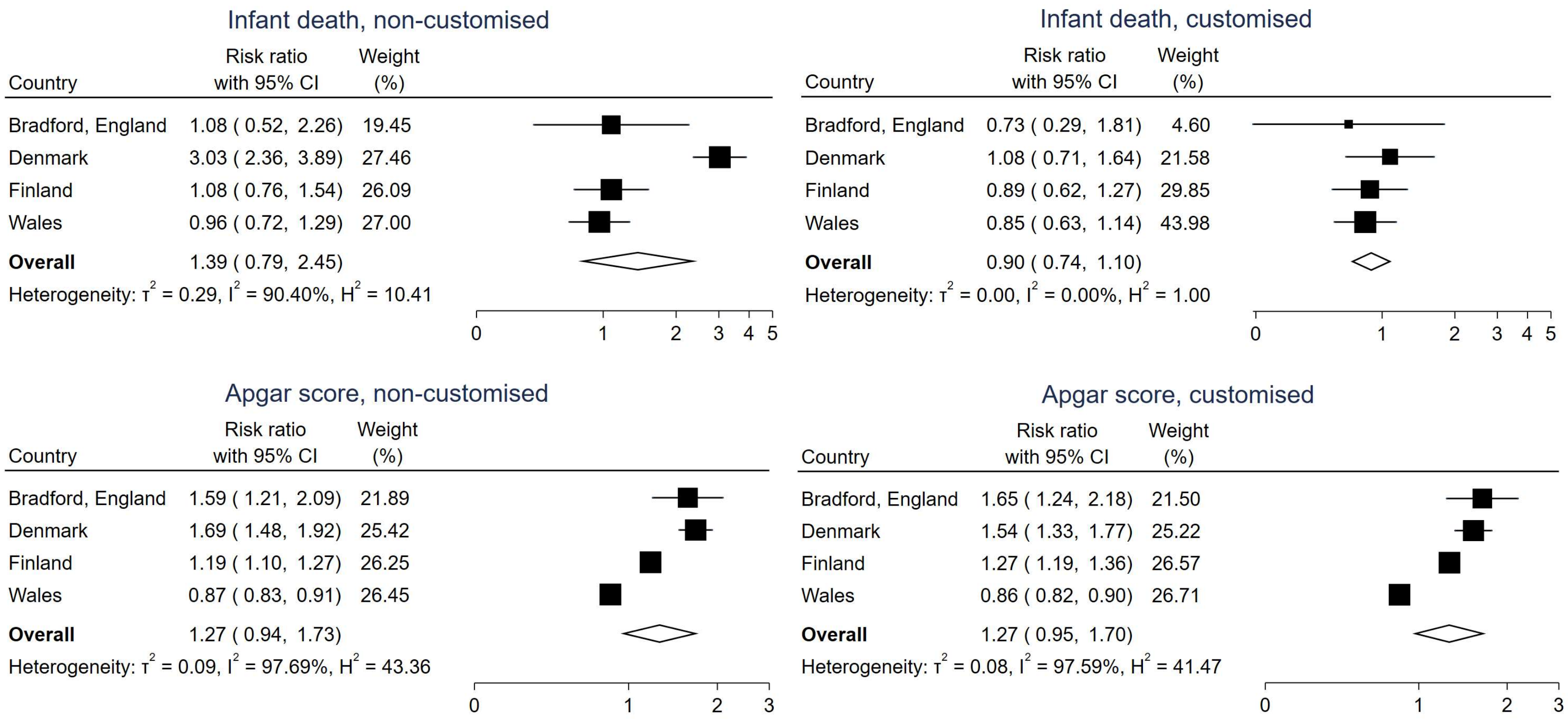
Infant death, non-customised
Country
Risk ratio with 95% CI
Weight (%)
Bradford, England 1.08 (0.52, 2.26) 19.45
Denmark 3.03 (2.36, 3.89) 27.46
Finland 1.08 (0.76, 1.54) 26.09
Wales 0.96 (0.72, 1.29) 27.00
Overall 1.39 (0.79, 2.45)
Heterogeneity: τ² = 0.29, I² = 90.40%, H² = 10.41
0 1 2 3 4 5
Infant death, customised
Country
Risk ratio with 95% CI
Weight (%)
Bradford, England 0.73 (0.29, 1.81) 4.60
Denmark 1.08 (0.71, 1.64) 21.58
Finland 0.89 (0.62, 1.27) 29.85
Wales 0.85 (0.63, 1.14) 43.98
Overall 0.90 (0.74, 1.10)
Heterogeneity: τ² = 0.00, I² = 0.00%, H² = 1.00
0 1 2 3 4 5
Apgar score, non-customised
Country
Risk ratio with 95% CI
Weight (%)
Bradford, England 1.59 (1.21, 2.09) 21.89
Denmark 1.69 (1.48, 1.92) 25.42
Finland 1.19 (1.10, 1.27) 26.25
Wales 0.87 (0.83, 0.91) 26.45
Overall 1.27 (0.94, 1.73)
Heterogeneity: τ² = 0.09, I² = 97.69%, H² = 43.36
0 1 2 3
Apgar score, customised
Country
Risk ratio with 95% CI
Weight (%)
Bradford, England 1.65 (1.24, 2.18) 21.50
Denmark 1.54 (1.33, 1.77) 25.22
Finland 1.27 (1.19, 1.36) 26.57
Wales 0.86 (0.82, 0.90) 26.71
Overall 1.27 (0.95, 1.70)
Heterogeneity: τ² = 0.08, I² = 97.59%, H² = 41.47
0 1 2 3

TABLE S1. Sample selection into main analyses

	Bradford	Denmark	Finland	Norway	Wales
Original sample of singleton births (N)	51,502	*434,322*	650,473	293,706	938,805
Sample after general exclusions [a] (N)	47,770	*385,356*	602,103	276,092*	845,228
Sample after variable restrictions [b] (N)	47,583	384,885	576,758	276,078	844,478

[a] General exclusions: major congenital anomaly (CA), infant sex, birthweight, or gestational age missing, maternal age ≤10, gestational age <37 or >43 weeks, birthweight <0.15kg or >8kg.
[b] Variable restrictions: maternal height accepted values 100-213cm, maternal weight accepted values 30-300kg, parity accepted range 0-15.
* Major CA not excluded.

TABLE S2 – Coefficients of birthweight centile customisation regression equations

	Bradford sensitivity analysis*	Denmark	Finland	Norway	Wales
Constant	3307.14	3477.51	3468.08	3484	3434.21
Residual standard error	409.48	420.19	403.27	401.95	433.93
Coefficients					
Sex**	-65.11	-67.82	-69.56	-56.14	-69.14
Gestational age	22.23	21.72	20.79	23.73	19.33
Ethnicity***	-171.08 (Pakistani)	-35.67 (Turkey)	27.77 (Russia)	-4.79 (Poland)	-107.90 (Indian)
Height	7.19	8.7	8.76	7.52	8.05
Weight	7.62	7.42	7.15	7.08	6.30
Weight quadratic	-0.11	-0.114	-0.12	-0.11	-0.07
Weight cubic	0	0	0	0	0
Parity 1	99.17	158.48	149.53	154.07	102.87
Parity 2	118.08	187.59	184.14	194.61	101.94
Parity 3	123.38	175.84	196.59	200.7	81.23
Parity 4 or more	130.69	171.76	234.09	200.65	57.95

* Customisation for main analysis performed with global bulk calculator from Perinatal Institute.
** Sex coded -1=male, 1=female, doubling this coefficient gives the difference between males and females. *** Example coefficient for largest minority group.

Kilpi F, *et al. BMJMED* 2023; 2:e000521. doi: 10.1136/bmjmed-2023-000521

TABLE S3A – BRADFORD Risks of adverse perinatal outcomes with different birthweight centile customization methods and centile cut-offs in Bradford

	Stillbirth		Neonatal death		Infant death		Apgar score at 5 min		NICU admission	
Birthweight thresholds	N (%)	Relative Risk	N (%)	Relative risk	N (%)	Relative risk	N (%)	Relative risk	N (%)	Relative risk
**Main analysis (full data)**										
Non-customised birthweight centiles										
<10th	30 (27)	3.60 (2.36- 5.49)	12 (26)	3.50 (1.80- 6.81)	25 (27)	3.77 (2.37- 6.00)	61 (14)	1.80 (1.37- 2.37)	334 (18)	2.48 (2.20- 2.79)
10-90th	75 (67)	1 (ref)	31 (66)	1 (ref)	60 (65)	1 (ref)	306 (72)	1 (ref)	1219 (66)	1 (ref)
>90th	7 (6)	0.76 (0.35- 1.64)	<5	1.04 (0.37- 2.96)	8 (9)	1.08 (0.52- 2.26)	60 (14)	1.59 (1.21- 2.09)	296 (16)	1.97 (1.74- 2.22)
<10th		3.70 (2.44- 5.61)		3.48 (1.81- 6.71)		3.74 (2.36- 5.90)		1.69 (1.29- 2.22)		2.24 (2.00-2.51)
>=10th		1 (ref)		1 (ref)		1 (ref)		1 (ref)		1 (ref)
Customised birthweight centiles										
<10th	31 (28)	2.39 (1.57- 3.62)	14 (30)	2.66 (1.41- 5.02)	30 (32)	2.95 (1.90- 4.59)	72 (17)	1.38 (1.07- 1.79)	416 (22)	2.04 (1.83- 2.27)
10-90th	74 (66)	1 (ref)	30 (64)	1 (ref)	58 (62)	1 (ref)	297 (70)	1 (ref)	1165 (63)	1 (ref)
>90th	7 (6)	0.80 (0.37- 1.73)	<5	0.84 (0.26- 2.76)	5 (5)	0.73 (0.29- 1.81)	58 (14)	1.65 (1.24- 2.18)	268 (14)	1.94 (1.70- 2.21)
<10th		2.44 (1.61- 3.68)		2.71 (1.45- 5.06)		3.04 (1.97- 4.69)		1.30 (1.01- 1.67)		1.85 (1.67- 2.06)
>=10th		1 (ref)		1 (ref)		1 (ref)		1 (ref)		1 (ref)
**Sensitivity analyses (complete case data for ethnicity)**										
Non-customised birthweight centiles										
<10th	29 (32)	4.42 (2.84- 6.88)	11 (28)	3.98 (1.96- 8.09)	23 (30)	4.43 (2.69- 7.29)	48 (16)	2.09 (1.53- 2.85)	255 (19)	2.62 (2.29- 3.00)
10-90th	59 (64)	1 (ref)	25 (64)	1 (ref)	47 (62)	1 (ref)	208 (71)	1 (ref)	881 (65)	1 (ref)
>90th	<5	0.55 (0.20- 1.51)	<5	0.97 (0.29- 3.22)	6 (8)	1.03 (0.44- 2.42)	38 (13)	1.48 (1.05- 2.09)	222 (16)	2.04 (1.77- 2.35)
<10th		4.65 (3.00- 7.21)		4.00 (1.99- 8.02)		4.41 (2.71- 7.19)		1.99 (1.46- 2.70)		2.35 (2.06-2.68)
>=10th		1 (ref)		1 (ref)		1 (ref)		1 (ref)		1 (ref)
Customised birthweight centiles (bulk calculator)										
<10th	28 (30)	3.03 (1.93- 4.75)	13 (33)	3.41 (1.74- 6.70)	25 (33)	3.35 (2.07- 5.44)	53 (18)	1.66 (1.23- 2.25)	293 (22)	2.19 (1.92- 2.49)
10-90th	58 (63)	1 (ref)	24 (62)	1 (ref)	47 (62)	1 (ref)	201 (68)	1 (ref)	844 (62)	1 (ref)

Kilpi F, *et al. BMJMED* 2023; 2:e000521. doi: 10.1136/bmjmed-2023-000521

>90th	6 (7)	0.77 (0.33- 1.79)	<5	0.62 (0.15- 2.63)	<5	0.63 (0.23- 1.76)	40 (14)	1.48 (1.06- 2.08)	221 (16)	1.95 (1.69- 2.26)
<10th		3.11 (2.00- 4.85)		3.57 (1.84- 6.95)		3.50 (2.17- 5.65)		1.57 (1.17- 2.11)		1.97 (1.74- 2.23)
>=10th		1 (ref)		1 (ref)		1 (ref)		1 (ref)		1 (ref)
Customised birthweight centiles (within-study customisation equation)										
<10th	29 (32)	3.95 (2.53-6.16)	12 (31)	3.82 (1.92-7.59)	24 (32)	3.98 (2.44-6.48)	46 (16)	1.73 (1.26-2.38)	281 (21)	2.56 (2.25-2.91)
10-90th	58 (63)	1 (ref)	25 (64)	1 (ref)	48 (63)	1 (ref)	211 (71)	1 (ref)	874 (64)	1 (ref)
>90th	5 (5)	0.77 (0.31-1.91)	<5	0.71 (0.17-3.01)	<5	0.74 (0.27-2.06)	38 (13)	1.56 (1.10-2.21)	203 (15)	2.07 (1.78-2.40)
<10th		4.05 (2.61-6.27)		3.93 (1.99-7.75)		4.08 (2.52-6.61)		1.64 (1.20-2.24)		2.31 (2.03-2.62)
>=10th		1 (ref)		1 (ref)		1 (ref)		1 (ref)		1 (ref)

TABLE S3B – DENMARK Risks of adverse perinatal outcomes with different birthweight centile customization methods and centile cut-offs in Denmark

	Stillbirth		Neonatal death		Infant death		Apgar score at 5 min	
Birthweight thresholds	N (%)	Relative Risk	N (%)	Relative risk	N (%)	Relative risk	N (%)	Relative risk
**Main analysis (full customisation, years 2004-2010)**								
Non-customised birthweight centiles								
<10th	142 (28)	3.77 (3.10-4.58)	38 (16)	2.83 (1.97-4.08)	57 (16)	2.30 (1.72-3.07)	283 (15)	1.94 (1.71-2.21)
10-90th	339 (66)	1 (ref)	121 (51)	1 (ref)	224 (61)	1 (ref)	1316 (70)	1 (ref)
>90th	35 (7)	0.83 (0.59-1.18)	77 (33)	5.14 (3.86-6.84)	84 (23)	3.03 (2.36-3.89)	275 (15)	1.69 (1.48-1.92)
<10th		3.84 (3.17-4.66)		1.95 (1.38-2.75)		1.88 (1.42-2.49)		1.81 (1.59-2.05)
>=10th		1 (ref)		1 (ref)		1 (ref)		1 (ref)
Customised birthweight centiles								
<10th	156 (30)	4.28 (3.54- 5.18)	98 (42)	7.37 (5.64-9.63)	123 (34)	5.00 (4.01- 6.24)	317 (17)	2.09 (1.85-2.36)
10-90th	322 (62)	1 (ref)	118 (50)	1 (ref)	218 (60)	1 (ref)	1346 (72)	1 (ref)
>90th	38 (8)	1.16 (0.83-1.62)	20 (8)	1.66 (1.03-2.66)	24 (7)	1.08 (0.71-1.64)	211 (11)	1.54 (1.33-1.77)
<10th		4.22 (3.50-5.09)		6.94 (5.36-8.99)		4.97 (4.00-6.17)		1.99 (1.77-2.25)
>=10th		1 (ref)		1 (ref)		1 (ref)		1 (ref)
**Sensitivity analysis (partial customisation for ethnicity and parity, years 1980-2010)**								
Non-customised birthweight centiles								
<10th	807 (31)	4.55 (4.18-4.95)	220 (19)	3.02 (2.59-3.52)	528 (18)	2.33 (2.12-2.57)	1431 (15)	1.86 (1.75-1.96)
10-90th	1598 (61)	1 (ref)	659 (58)	1 (ref)	2045 (70)	1 (ref)	6987 (73)	1 (ref)
>90th	211 (8)	1.07 (0.93-1.24)	255 (22)	3.14 (2.72-3.63)	358 (12)	1.42 (1.27-1.59)	1213 (13)	1.41 (1.33-1.50)
<10th		4.51 (4.15; 4.90)		2.44 (2.11; 2.83)		2.23 (2.03-2.45)		1.78 (1.68-1.88)
>=10th		1 (ref)		1 (ref)		1 (ref)		1 (ref)
Customised birthweight centiles								
<10th	812 (31)	4.90 (4.51-5.33)	377 (33)	5.70 (5.02-6.47)	706 (24)	3.40 (3.12-3.71)	1438 (15)	1.99 (1.88-2.10)
10-90th	1609 (62)	1 (ref)	646 (57)	1 (ref)	2025 (69)	1 (ref)	7082 (74)	1 (ref)
>90th	195 (7)	1.06 (0.92-1.23)	111 (10)	1.51 (1.23-1.84)	200 (7)	0.87 (0.75-1.00)	1111 (12)	1.38 (1.29-1.47)

<10th		4.87 (4.49-5.29)		5.41 (4.79-6.13)		3.45 (3.17-3.75)		1.91 (1.81-2.02)
>=10th		1 (ref)		1 (ref)		1 (ref)		1 (ref)

TABLE S3C – FINLAND Risks of adverse perinatal outcomes with different birthweight centile customization methods and centile cut-offs in Finland

	Stillbirth		Neonatal death		Infant death		Apgar score at 5 min	
Birthweight thresholds	N (%)	Relative Risk	N (%)	Relative risk	N (%)	Relative risk	N (%)	Relative risk
Non-customised birthweight centiles								
<10th	144 (27)	3.61 (2.97- 4.38)	23 (17)	2.05 (1.30- 3.22)	53 (15)	1.81 (1.35- 2.43)	992 (13)	1.60 (1.50- 1.71)
10-90th	358 (67)	1 (ref)	101 (73)	1 (ref)	263 (75)	1 (ref)	5656 (75)	1 (ref)
>90th	36 (7)	0.82 (0.58- 1.15)	15 (11)	1.21 (0.70- 2.07)	35 (10)	1.08 (0.76- 1.54)	850 (11)	1.19 (1.10- 1.27)
<10th		3.68 (3.04- 4.46)		2.00 (1.28- 3.13)		1.80 (1.34- 2.40)		1.57 (1.47- 1.68)
>=10th		1 (ref)		1 (ref)		1 (ref)		1 (ref)
Customised birthweight centiles								
<10th	169 (31)	3.31 (2.75- 3.98)	31 (22)	2.11 (1.41- 3.17)	69 (20)	1.80 (1.38- 2.35)	881 (12)	1.34 (1.25- 1.44)
10-90th	330 (61)	1 (ref)	95 (68)	1 (ref)	248 (71)	1 (ref)	5519 (74)	1 (ref)
>90th	39 (7)	0.77 (0.55- 1.07)	13 (9)	0.89 (0.50- 1.58)	34 (10)	0.89 (0.62- 1.27)	1098 (15)	1.27 (1.19- 1.36)
<10th		3.41 (2.85- 4.10)		2.14 (1.44- 3.20)		1.83 (1.40- 2.38)		1.30 (1.21- 1.39)
>=10th		1 (ref)		1 (ref)		1 (ref)		1 (ref)

Kilpi F, *et al. BMJMED* 2023; 2:e000521. doi: 10.1136/bmjmed-2023-000521

TABLE S3D – NORWAY Risks of adverse perinatal outcomes with different birthweight centile customization methods and centile cut-offs in Norway

	Stillbirth		Neonatal death		Infant death		Apgar score at 5 min	
Birthweight thresholds	N (%)	Relative Risk	N (%)	Relative risk	N (%)	Relative risk	N (%)	Relative risk
**Main analysis (full customisation, years 2012-2016)**								
Non-customised birthweight centiles								
<10th	82 (22)	2.94 (2.29-3.77)	38 (29)	4.18 (2.85-6.14)	73 (30)	4.30 (3.26-5.68)	378 (14)	1.72 (1.54-1.92)
10-90th	251 (68)	1 (ref)	82 (64)	1 (ref)	153 (63)	1 (ref)	1983 (73)	1 (ref)
>90th	37 (10)	1.19 (0.85-1.69)	9 (7)	0.89 (0.45-1.77)	16 (7)	0.85 (0.51-1.42)	338 (13)	1.38 (1.23-1.55)
<10th		2.88 (2.25-3.68)		4.23 (2.90-6.18)		4.38 (3.33-5.76)		1.65 (1.48-1.84)
>=10th		1 (ref)		1 (ref)		1 (ref)		1 (ref)
Customised birthweight centiles								
<10th	94 (25)	3.21 (2.53-4.07)	39 (30)	3.96 (2.71-5.79)	77 (32)	4.33 (3.29-5.69)	375 (14)	1.59 (1.43-1.78)
10-90th	246 (66)	1 (ref)	83 (64)	1 (ref)	150 (62)	1 (ref)	1987 (74)	1 (ref)
>90th	30 (8)	1.13 (0.78-1.66)	7 (5)	0.78 (0.36-1.70)	15 (6)	0.93 (0.55-1.58)	337 (12)	1.58 (1.41-1.77)
<10th		3.17 (2.51-4.01)		4.05 (2.78-5.89)		4.36 (3.32-5.71)		1.51 (1.35-1.68)
>=10th		1 (ref)		1 (ref)		1 (ref)		1 (ref)
**Sensitivity analysis (partial customisation for ethnicity and parity, years 1999-2016)**								
Non-customised birthweight centiles								
<10th	420 (28)	3.98 (3.55-4.47)	163 (26)	3.60 (3.00-4.31)	315 (26)	3.56 (3.13-4.06)	1315 (14)	1.77 (1.67-1.88)
10-90th	949 (62)	1 (ref)	409 (65)	1 (ref)	798 (66)	1 (ref)	6713 (73)	1 (ref)
>90th	155 (10)	1.32 (1.12-1.57)	57 (9)	1.13 (0.86-1.49)	88 (7)	0.89 (0.72- 1.11)	1172 (13)	1.41 (1.33-1.50)
<10th		3.85 (3.44-4.30)		3.55 (2.97-4.24)		3.61 (3.17- 4.10)		1.69 (1.60-1.79)
>=10th		1 (ref)		1 (ref)		1 (ref)		1 (ref)
Customised birthweight centiles								
<10th	434 (28)	4.46 (3.98-4.99)	155 (25)	5.71 (5.03-6.47)	309 (26)	3.72 (3.27-4.24)	1111 (12)	1.58 (1.48-1.68)
10-90th	944 (62)	1 (ref)	417 (66)	1 (ref)	808 (67)	1 (ref)	6861 (75)	1 (ref)
>90th	146 (10)	1.38 (1.16-1.64)	57 (9)	1.50 (1.23-1.84)	84 (7)	0.93 (0.74-1.16)	1228 (13)	1.60 (1.50-1.69)

<10th		4.29 (3.84-4.80)		5.43 (4.80-6.13)		3.75 (3.30-4.27)		1.49 (1.40-1.58)
>=10th		1 (ref)		1 (ref)		1 (ref)		1 (ref)

TABLE S3E – WALES Risks of adverse perinatal outcomes with different birthweight centile customization methods and centile cut-offs in Wales

	Stillbirth		Neonatal death		Infant death		Apgar score at 5 min	
Birthweight thresholds	N (%)	Relative Risk	N (%)	Relative risk	N (%)	Relative risk	N (%)	Relative risk
**Main analysis (full data, years 1987-2016)**								
Non-customised birthweight centiles								
<10th	292 (26)	3.72 (3.25-4.27)	45 (18)	2.32 (1.67-3.22)	135 (22)	2.78 (2.29-3.37)	2,357 (12)	1.43 (1.37-1.49)
10-90th	705 (64)	1 (ref)	175 (72)	1 (ref)	438 (70)	1 (ref)	15,046 (79)	1 (ref)
>90th	105 (10)	1.21 (0.99-1.48)	24 (10)	1.11 (0.73-1.71)	52 (8)	0.96 (0.72-1.29)	1,627 (9)	0.87 (0.83-0.91)
<10th		3.64 (3.19-4.16)		2.29 (1.66-3.16)		2.79 (2.31-3.38)		1.45 (1.39-1.52)
>=10th		1 (ref)		1 (ref)		1 (ref)		1 (ref)
Customised birthweight centiles								
<10th	326 (30)	4.21 (3.69-4.81)	45 (18)	2.24 (1.61-3.11)	138 (22)	2.73 (2.25-3.30)	2,335 (12)	1.38 (1.32-1.44)
10-90th	668 (61)	1 (ref)	174 (71)	1 (ref)	438 (70)	1 (ref)	14,995 (79)	1 (ref)
>90th	108 (10)	1.23 (1.00-1.51)	25 (10)	1.09 (0.72-1.66)	49 (8)	0.85 (0.63-1.14)	1,700 (9)	0.86 (0.82-0.90)
<10th		4.10 (3.60-4.67)		2.21 (1.60-3.06)		2.78 (2.30-3.35)		1.40 (1.34-1.46)
>=10th		1 (ref)		1 (ref)		1 (ref)		1 (ref)
**Sensitivity analysis (restricted data, years 2003-2016)**								
Non-customised birthweight centiles								
<10th	185 (27)	3.79 (3.19-4.50)	38 (19)	2.45 (1.71-3.50)	88 (21)	2.68 (2.11-3.40)	421 (13)	1.60 (1.44-1.77)
10-90th	438 (64)	1 (ref)	140 (71)	1 (ref)	296 (72)	1 (ref)	2371 (75)	1 (ref)
>90th	60 (9)	1.10 (0.84-1.44)	18 (9)	1.03 (0.63-1.69)	29 (7)	0.79 (0.54-1.15)	351 (11)	1.20 (1.07-1.34)
<10th		3.75 (3.17-4.44)		2.44 (1.71-3.47)		2.74 (2.17-3.47)		1.56 (1.41-1.73)
>=10th		1 (ref)		1 (ref)		1 (ref)		1 (ref)
Customised birthweight centiles								
<10th	201 (29)	4.28 (3.62-5.07)	37 (19)	2.36 (1.64-3.39)	87 (21)	2.65 (2.09-3.37)	398 (13)	1.50 (1.35-1.67)
10-90th	417 (61)	1 (ref)	140 (19)	1 (ref)	293 (71)	1 (ref)	2380 (76)	1 (ref)
>90th	65 (10)	1.21 (0.93-1.57)	19 (10)	1.06 (0.65-1.70)	33 (8)	0.88 (0.61-1.26)	365 (12)	1.20 (1.07-1.34)
<10th		4.18 (3.55-4.93)		2.34 (1.64-3.35)		2.69 (2.12-3.40)		1.47 (1.32-1.63)

>=10th		1 (ref)		1 (ref)		1 (ref)		1 (ref)

TABLE S4 – Pooled estimates of sensitivity and specificity of SGA and LGA versus AGA (<10th or >90th vs 10-90th) for adverse perinatal outcomes by non-customised and customised birthweight centiles

	Non-customised		Customised	
Outcome	Sensitivity	Specificity	Sensitivity	Specificity
Stillbirth	0.34 (0.33-0.36)	0.82 (0.81-0.84)	0.38 (0.36-0.40)	0.80 (0.78-0.82)
Neonatal death	0.34 (0.27-0.42)	0.82 (0.81-0.84)	0.36 (0.29-0.43)	0.80 (0.78-0.82)
Infant death	0.31 (0.27-0.36)	0.82 (0.81-0.84)	0.34 (0.29-0.38)	0.80 (0.78-0.82)
Apgar score	0.26 (0.23-0.29)	0.82 (0.81-0.83)	0.26 (0.23-0.29)	0.80 (0.78-0.82)

Kilpi F, *et al. BMJMED* 2023; 2:e000521. doi: 10.1136/bmjmed-2023-000521

TABLE S5A – BRADFORD Sensitivity and specificity of different birthweight centile cut-offs in Bradford

		Stillbirth		Neonatal death		Infant death		Apgar score		NICU admission	
Birthweight thresholds		Sens	Spec	Sens	Spec	Sens	Spec	Sens	Spec	Sens	Spec
**Main analysis (full data)**											
Non-customised centiles											
	10/90th	0.33	0.81	0.34	0.82	0.36	0.81	0.28	0.81	0.34	0.82
	10th	0.27	0.91	0.18	0.91	0.27	0.91	0.14	0.91	0.18	0.91
Customised centiles (bulk calculator)											
	10/90th	0.34	0.77	0.36	0.77	0.38	0.77	0.30	0.77	0.37	0.78
	10th	0.28	0.87	0.30	0.86	0.32	0.87	0.17	0.87	0.23	0.87
**Sensitivity analyses (complete case data for ethnicity)**											
Non-customised centiles											
	10/90th	0.36	0.81	0.36	0.81	0.38	0.81	0.29	0.81	0.35	0.82
	10th	0.32	0.91	0.28	0.91	0.30	0.91	0.16	0.91	0.19	0.91
Customised centiles (bulk calculator)											
	10/90th	0.37	0.77	0.39	0.77	0.38	0.77	0.32	0.77	0.38	0.78
	10th	0.30	0.88	0.33	0.88	0.33	0.88	0.18	0.88	0.22	0.88
Customised centiles (within-study customisation with equation)											
	10/90th	0.37	0.81	0.36	0.81	0.37	0.81	0.28	0.81	0.36	0.81
	10th	0.32	0.90	0.31	0.90	0.32	0.90	0.16	0.90	0.21	0.90

Kilpi F, *et al. BMJMED* 2023; 2:e000521. doi: 10.1136/bmjmed-2023-000521

TABLE S5B – DENMARK Sensitivity and specificity of different birthweight centile cut-offs in Denmark

		Stillbirth		Neonatal death		Infant death		Apgar score	
Birthweight thresholds		Sens	Spec	Sens	Spec	Sens	Spec	Sens	Spec
**Main analysis (full customisation, years 2004-2010)**									
Non-customised centiles									
	10/90th	0.34	0.81	0.49	0.81	0.39	0.81	0.30	0.81
	10th	0.28	0.91	0.16	0.91	0.16	0.91	0.15	0.91
Customised centiles									
	10/90th	0.38	0.82	0.50	0.82	0.40	0.82	0.28	0.82
	10th	0.30	0.91	0.42	0.91	0.34	0.91	0.17	0.91
**Sensitivity analysis (partial customisation for ethnicity and parity, years 1980-2010)**									
Non-customised centiles									
	10/90th	0.39	0.81	0.42	0.81	0.30	0.81	0.28	0.81
	10th	0.31	0.91	0.19	0.91	0.18	0.91	0.15	0.91
Customised centiles									
	10/90th	0.39	0.82	0.43	0.82	0.31	0.82	0.27	0.82
	10th	0.31	0.92	0.33	0.92	0.24	0.92	0.15	0.92

Kilpi F, *et al. BMJMED* 2023; 2:e000521. doi: 10.1136/bmjmed-2023-000521

TABLE S5C – FINLAND Sensitivity and specificity of different birthweight centile cut-offs in Finland

		Stillbirth		Neonatal death		Infant death		Apgar score	
Birthweight thresholds		Sens	Spec	Sens	Spec	Sens	Spec	Sens	Spec
**Main analysis**									
Non-customised centiles									
	10/90th	0.34	0.81	0.27	0.81	0.25	0.81	0.25	0.81
	10th	0.27	0.91	0.17	0.91	0.15	0.91	0.14	0.91
Customised centiles									
	10/90th	0.39	0.76	0.32	0.76	0.29	0.76	0.27	0.79
	10th	0.31	0.88	0.22	0.88	0.20	0.88	0.12	0.91

Kilpi F, *et al. BMJMED* 2023; 2:e000521. doi: 10.1136/bmjmed-2023-000521

TABLE S5D – NORWAY Sensitivity and specificity of different birthweight centile cut-offs in Norway

		Stillbirth		Neonatal death		Infant death		Apgar score	
Birthweight thresholds		Sens	Spec	Sens	Spec	Sens	Spec	Sens	Spec
**Main analysis (full customisation, years 2012-2016)**									
Non-customised centiles									
	10/90th	0.32	0.81	0.36	0.81	0.37	0.81	0.27	0.81
	10th	0.22	0.91	0.30	0.91	0.30	0.91	0.14	0.91
Customised centiles									
	10/90th	0.34	0.82	0.36	0.82	0.38	0.82	0.26	0.82
	10th	0.25	0.90	0.30	0.90	0.32	0.90	0.14	0.90
**Sensitivity analysis (partial customisation for ethnicity and parity, years 1999-2016)**									
Non-customised centiles									
	10/90th	0.38	0.81	0.35	0.81	0.34	0.81	0.27	0.81
	10th	0.28	0.91	0.26	0.91	0.26	0.91	0.14	0.91
Customised centiles									
	10/90th	0.38	0.82	0.34	0.82	0.33	0.82	0.25	0.82
	10th	0.29	0.92	0.25	0.92	0.26	0.92	0.12	0.92

TABLE S5E – WALES Sensitivity and specificity of different birthweight centile cut-offs in Wales

		Stillbirth		Neonatal death		Infant death		Apgar score	
Birthweight thresholds		Sens	Spec	Sens	Spec	Sens	Spec	Sens	Spec
**Main analysis (full data, years 1987-2016)**									
Non-customised centiles									
	10/90th	0.36	0.81	0.28	0.81	0.30	0.81	0.21	0.81
	10th	0.27	0.91	0.18	0.91	0.22	0.91	0.12	0.91
Customised centiles									
	10/90th	0.39	0.80	0.29	0.80	0.30	0.80	0.21	0.80
	10th	0.30	0.91	0.18	0.91	0.22	0.91	0.12	0.91
**Sensitivity analysis (restricted data, years 2003-2016)**									
Non-customised centiles									
	10/90th	0.36	0.81	0.29	0.81	0.28	0.81	0.25	0.81
	10th	0.27	0.91	0.19	0.91	0.21	0.91	0.13	0.91
Customised centiles									
	10/90th	0.39	0.81	0.29	0.81	0.29	0.81	0.24	0.81
	10th	0.29	0.91	0.19	0.91	0.21	0.91	0.13	0.91

TABLE S6 – Bradford ethnicity categorisation

	Code in bulk calculator	N (%)	Code in bulk calculator	Customisation coefficient
Code in Bradford data	Main analysis<br>(N=47,583)		Sensitivity analysis	
African	SSA (Sub-Saharan African)	563 (1.60)	SSA (Sub-Saharan African)	-125.70
Bangladeshi	BGD	1,225 (3.47)	BGD	-170.35
British	ENG	14,163 (40.13)	ENG	(reference)
Caribbean	CAR	121 (0.34)	CAR	-148.60
Chinese	CHN	90 (0.26)	CHN	NA
Indian	IND	1,081 (3.06)	IND	-162.40
Irish	IRL	60 (0.17)	IRL	-164.37
Pakistani	PAK	17,986 (50.97)	PAK	-171.08
	Global average	12,294 (25.84)		
Any other Asian	Global average		Not included	
Any other Black	Global average		Not included	
Any other White	Global average		Not included	
Any other ethnic group	Global average		Not included	
Any other mixed background	Global average		Not included	
Not stated	Global average		Not included	
White and Asian	Global average		Not included	
White and Black African	Global average		Not included	
White and Black Caribbean	Global average		Not included	
Missing	Global average		Not included	

Kilpi F, *et al. BMJMED* 2023; 2:e000521. doi: 10.1136/bmjmed-2023-000521

TABLE S7 – Denmark maternal country of origin frequencies and customisation coefficients

	N	%	Customisation coefficient
Denmark	330315	86.04%	(reference)
Afghanistan	1458	0.38%	43.47
Algeria	129	0.03%	0.35
Australia	131	0.03%	41.41
Belarus	113	0.03%	52.65
Bosnia-Herzegovina	1583	0.41%	52.92
Brazil	371	0.10%	41.3
Bulgaria	198	0.05%	-0.94
Burundi	110	0.03%	-129.52
Canada	150	0.04%	-11.51
Caribbean	133	0.03%	33.79
Chile	138	0.04%	111.36
Eastern Europe	847	0.22%	0.15
Egypt	152	0.04%	-47.49
Estonia	183	0.05%	50.77
Ethiopia	121	0.03%	-11.34
Far East Asia	118	0.03%	64.85
Philippines	989	0.26%	151.27
Finland	337	0.09%	23.24
France	349	0.09%	-25.94
Ghana	208	0.05%	-134.24
India	439	0.11%	-107.83
Indonesia	171	0.04%	94.43
Iraq	3382	0.88%	-46.63
Iran	930	0.24%	-3.28
Iceland	1254	0.33%	73.23
Israel	143	0.04%	-112.63
Italy	186	0.05%	-34.57
Japan	166	0.04%	-30.84
Jordan	330	0.09%	-86.69
Yugoslavia	1472	0.38%	-27.57
Kenya	118	0.03%	-59.92
China	875	0.23%	160.42
Kuwait	251	0.07%	-104.55
Latvia	326	0.08%	84.75
Lebanon	2764	0.72%	-84.58
Lithuania	661	0.17%	70.16
Morocco	1379	0.36%	7.37
Middle East	141	0.04%	-63.14
Myanmar	188	0.05%	47.48
Netherlands	538	0.14%	0.15
Nigeria	138	0.04%	7.25
North Macedonia	447	0.12%	-26.87

Norway	1729	0.45%	2.82
Pakistan	2773	0.72%	-132.97
Peru	178	0.05%	216.39
Poland	1905	0.50%	-10.5
Romania	490	0.13%	45.34
Russia	753	0.20%	61.24
Serbia Montenegro	191	0.05%	17.48
Somalia	3202	0.83%	-173.27
South America	328	0.09%	86.63
South Asia	132	0.03%	-52.09
South East Asia	114	0.03%	9.1
South Europe	113	0.03%	-21.56
Spain	226	0.06%	26.92
Sri Lanka	913	0.24%	-42.93
Great Britain	682	0.18%	-8.07
Sub Saharan Africa	864	0.23%	-46.85
Sudan	106	0.03%	-205.21
Sweden	1375	0.36%	16.06
Syria	505	0.13%	-67.49
Tanzania	126	0.03%	-14.77
Thailand	1325	0.35%	157.42
Tunisia	127	0.03%	-94.05
Turkey	6489	1.69%	-35.67
Germany	1596	0.42%	-42.43
USA	490	0.13%	26.59
Uganda	172	0.04%	-53.65
Ukraine	620	0.16%	91.14
Hungary	176	0.05%	12.14
Vietnam	1464	0.38%	38.71
West Europe	328	0.09%	-41.12

Table S8 – Finland maternal country of birth frequencies and customisation coefficients

Country	N	%	Customisation coefficient
Finland	523643	90.79	(reference)
Afghanistan	777	0.13	17.21
Algeria	181	0.03	-79.88
Angola	145	0.03	-156.96
Australia	114	0.02	-32.28
Bangladesh	340	0.06	-185.2
Bosnia-Herzegovina	126	0.02	55.9
Brazil	299	0.05	-5.83
Bulgaria	201	0.03	-52.59
Myanmar	200	0.03	-46.77
Cameroon	151	0.03	-16.54
Canada	244	0.04	-41.15
Sri Lanka	184	0.03	-77.3
China	1376	0.24	96.42
Columbia	121	0.02	-3.31
Congo	393	0.07	-133.68
Former Czechoslovakia	105	0.02	15.22
Ethiopia	329	0.06	-64.19
Estonia	4587	0.8	37.2
France	262	0.05	-31.23
Germany	759	0.13	-39.05
Ghana	250	0.04	-158.07
Hungary	290	0.05	-24.66
India	716	0.12	-160.7
Indonesia	122	0.02	55.46
Iran	655	0.11	-26.05
Iraq	1972	0.34	-91.26
Italia	191	0.03	-63.18
Japan	323	0.06	-60.79
Kenya	292	0.05	-75.41
Latvia	277	0.05	-0.35
Lithuania	170	0.03	62.51
Mexico	110	0.02	22.26
Morocco	522	0.09	-26.88
Nepal	126	0.02	-88.02
Netherlands	104	0.02	-20.96
Nigeria	314	0.05	-81.88
Norway	173	0.03	7.39
Pakistan	318	0.06	-170.89
Philippines	597	0.1	119.22
Poland	536	0.09	0.04
Romania	337	0.06	3.09
Russia	9825	1.7	27.77
Vietnam	1267	0.22	3.23

Kilpi F, *et al. BMJMED* 2023; 2:e000521. doi: 10.1136/bmjmed-2023-000521

Somalia	3462	0.6	-207.03
Spain	242	0.04	-1.42
Former Sudan	390	0.07	-265.97
Sweden	8570	1.49	1.09
Syria	151	0.03	-161.1
Thailand	2064	0.36	137.46
Tunisia	132	0.02	-57.41
Turkey	1098	0.19	-105.69
Ukraine	207	0.04	39.86
United Kingdom	301	0.05	-15.74
Tanzania	114	0.02	-26.46
USA	502	0.09	-15.51
Former Yugoslavia	1953	0.34	-43.48
Former Serbia & Montenegro	259	0.04	10.58
Zambia	540	0.09	-24.4
East Europe	326	0.06	-17.26
Caribbean	107	0.02	1.53
South America	404	0.07	60.82
Middle East	355	0.06	-108.19
West Europe	245	0.04	-49.58
Sub Saharan Africa	430	0.07	-92.52
Pacific Islands	143	0.02	42.67
South East Asia	156	0.03	-10.55

Kilpi F, *et al. BMJMED* 2023; 2:e000521. doi: 10.1136/bmjmed-2023-000521

TABLE S9 – Norway maternal country of birth frequencies and customisation coefficients

Country/region	N	%	Customisation coefficient
Norway	196642	71.23	(reference)
Denmark	1143	0.41	-10.91
Finland	492	0.18	31.74
Iceland	633	0.23	40.41
Sweden	4.574	1.66	20.72
Albania	161	0.06	31.11
Belgium	102	0.04	-39.64
Bulgaria	520	0.19	-9.73
Estonia	429	0.16	-5.30
France	491	0.18	-49.70
Greece	130	0.05	-67.51
Belarus	195	0.07	96.58
Croatia	210	0.08	-18.17
Italy	226	0.08	-76.85
Latvia	941	0.34	84.07
Serbia & Montenegro	993	0.36	4.03
Netherlands	426	0.15	-5.81
Poland	7696	2.79	-4.79
Portugal	209	0.08	-179.10
Romania	1472	0.53	-11.85
Lithuania	4247	1.54	28.58
Spain	394	0.14	-123.34
Moldova	134	0.05	78.40
United Kingdom	796	0.29	-30.43
Russia	2233	0.81	41.99
Switzerland	166	0.06	-83.10
Turkey	1145	0.41	-49.86
Germany	1976	0.72	-68.50
Ukraine	770	0.28	11.57
Hungary	325	0.12	-43.45
Austria	118	0.04	2.43
Bosnia / Herzegovina	933	0.34	-22.84
North Macedonia	215	0.08	-97.69
Slovakia	393	0.14	-35.55
Czechia	237	0.09	-115.03
Serbia	455	0.16	-18.26
Kosovo	520	0.19	-36.78
Algeria	141	0.05	-95.60
Burundi	150	0.05	-44.26
Eritrea	2048	0.74	-57.85
Ethiopia	1365	0.49	-79.65
Gambia	116	0.04	-132.34
Ghana	254	0.09	-186.86

Cameroon	105	0.04	-11.08
Kenya	277	0.10	-64.24
Congo	284	0.10	-93.62
Liberia	118	0.04	-74.42
Morocco	749	0.27	-24.24
Nigeria	272	0.10	-34.68
Somalia	5064	1.83	-226.70
Sudan	505	0.18	-191.46
South Africa	101	0.04	-81.03
Tanzania	103	0.04	10.62
Tunisia	129	0.05	-47.58
Uganda	156	0.06	-60.98
Afghanistan	1646	0.60	-1.60
Myanmar	289	0.10	4.95
Sri Lanka	793	0.29	-107.24
Philippines	2984	1.08	130.84
India	1169	0.42	-121.33
Indonesia	254	0.09	64.68
Iraq	3048	1.10	-54.04
Iran	1251	0.45	-33.33
Japan	152	0.06	9.52
Kazakhstan	136	0.05	10.38
China	1008	0.39	60.42
Korea	817	0.30	64.75
Lebanon	184	0.07	-24.46
Palestine	341	0.12	-128.64
Nepal	164	0.06	-1.74
Pakistan	2452	0.89	-174.08
Saudia Arabia	124	0.04	-256.38
Syria	797	0.29	-164.17
Thailand	2012	0.73	119.20
Vietnam	1407	0.51	27.97
Canada	227	0.08	-27.69
Cuba	106	0.04	-103.27
Dominican Republic	103	0.04	-54.31
Mexico	163	0.06	-13.12
USA	839	0.30	-23.44
Argentina	105	0.04	21.02
Brazil	826	0.30	0.33
Chile	380	0.14	5.73
Colombia	532	0.19	18.32
Peru	221	0.08	145.09
Venezuela	122	0.04	-43.06
Australia	168	0.06	-26.81
East Europe	210	0.08	-0.51
Sub Saharan Africa	621	0.22	-127.35

Far East Asia	147	0.05	46.60
Central Asia	114	0.04	50.97
South America	332	0.12	37.65
Other	1.068	0.39	-71.19
Unspecified or stateless or unknown categories	4015	1.45	Not included

Kilpi F, *et al. BMJMED* 2023; 2:e000521. doi: 10.1136/bmjmed-2023-000521

TABLE S10 - Welsh ethnicity codes and frequencies

We used the current NHS Wales Data Dictionary codes (those in use since 1st April 2002) as the key (see below) to categorise the ethnicity variable in the data. The other codes available in the SAIL databank data were deemed too unreliable to use.

**Code**	**Category**	Frequency in main analysis %
A	Any White Background	43.03
D	White and Black Caribbean	0.4
E	White and Black African	0.16
F	White and Asian	0.22
G	Any other mixed background	0.42
H	Indian	0.39
J	Pakistani	0.32
K	Bangladeshi	0.37
L	Any other Asian background	0.28
M	Caribbean	0.09
N	African	0.3
P	Any other Black background	0.14
R	Chinese	0.09
S	Any other ethnic group	0.37
	Other codes or missing	53.43

Kilpi F, *et al. BMJMED* 2023; 2:e000521. doi: 10.1136/bmjmed-2023-000521
